# TiO_2_ decorated functionalized halloysite nanotubes (TiO_2_@HNTs) and photocatalytic PVC membranes synthesis, characterization and its application in water treatment

**DOI:** 10.1038/s41598-019-40775-4

**Published:** 2019-03-13

**Authors:** Gourav Mishra, Mausumi Mukhopadhyay

**Affiliations:** Department of Chemical Engineering, Sardar Vallabhbhai National Institute of Technology Surat, Gujarat, India

## Abstract

In this study photocatalyst, TiO_2_@HNTs were prepared by  synthesizing TiO_2_ nanoparticles *in situ* on the  functionalized halloysite nanotubes (HNTs) surface. Photocatalytic PVC membrane TiO_2_@HNTs M2 (2 wt.%) and TiO_2_@HNTs M3 (3 wt.%) were also prepared. Photocatalyst TiO_2_@HNTs and photocatalytic PVC membranes were used to study the photocatalytic activity against the methylene blue (MB) and rhodamine B (RB) dyes in UV batch reactor. The structure and morphology of photocatalyst and photocatalytic PVC membrane were characterized by fourier transform infrared spectroscopy (FT-IR), X-ray diffraction (XRD), scanning electron microscopy (SEM), energy dispersive X-ray (EDX), transmission electron microscopy (TEM), UV-Vis spectrophotometer and photoluminescence (PL). The PL study showed that the oxygen vacancies and surface hydroxyl groups present on the surface of TiO_2_@HNTs act as excellent traps for charge carrier, reducing the electron-hole recombination rate.TiO_2_@HNTs 2 (2 wt.%) and TiO_2_@HNTs 3 (3 wt.%) degraded MB dye up to 83.21%, 87.47% and RB dye up to 96.84% and 96.87%, respectively. TiO_2_@HNT photocatalyst proved to be stable during the three consecutive cycle of photocatalytic degradation of the RB dye. TiO_2_@HNTs M2 and TiO_2_@HNTs M3 degraded MB dye up to 27.19%, 42.37% and RB dye up to 30.78%, 32.76%, respectively. Photocatalytic degradation of both the dyes followed the first-order kinetic model. Degradation product analysis was done using the liquid chromatography–mass spectrometry (LC-MS) and the results showed that the dye degradation was initiated by demethylation of the molecule. MB and RB dye degradation reaction were tested by TBA and IPA as OH^*^ and H^+^ scavengers respectively. Mechanism of photocatalytic activity of TiO_2_@HNTs and photocatalytic PVC membrane were also explained.

## Introduction

In recent years, halloysite nanotubes (HNTs) and its composite materials have attracted enormous attention due to their wide range of potential applications in fields of catalysis^1^, adsorption^2^, composites^3^ and drug delivery processes^4^ etc. Various materials like HNTs, carbon nanotubes (CNTs), graphene oxide (GO), silica, cerium oxide^5^ and clay particles have been attached to photocatalysts^1^ to improve the photocatalytic reactions. Amongst them, HNTs (Al_2_Si_2_O_5_(OH)_4_·2H_2_O) is a promising material due to its efficient physicochemical property and stability which has been tested in various studies^6–9^. HNTs are naturally occurring, eco-friendly alumino-silicate clay minerals with tube-like structure, consisting of two-layered aluminosilicate clay mineral with one alumina octahedron sheet and one silica tetrahedron sheet in 1:1 stoichiometric ratio^10,11^. They are suitable for the development of photocatalyst supported materials as they enable proper distribution of composite in suspensions. The presence of HNTs improves the synergistic effects and light absorption properties of photocatalyst supported materials. Hydroxyl groups found on its surface helps in better dispersion of HNTs in different solvents^12^. Comparatively, HNTs are more efficient than carbon nanotubes (CNTs) due to many –OH groups present on its surface and different outside and inside chemical properties. The fact that HNTs are structurally similar to CNTs but cheaper than CNTs makes them ideal for research in photocatalysis and adsorption.

Photocatalysts are semiconductor that converts light energy into chemical energy of electron-hole pairs. An efficient photocatalyst must possess chemical and physical stability, must be cheap and nontoxic in nature^13^. Several semiconductor materials such as TiO_2_, titanate nanosheets^14^, cobalt hydroxide-amino complex^15^, ZnO^16^, CdS, g-C_3_N_4_ and Ag_3_PO_4_, and their nanostructure assemblies have been extensively employed as photocatalysts^17^.

TiO_2_ (nano range) has been greatly used in photocatalytic applications due to its exceptional properties such as quantum confinement and high surface to volume ratio^18–20^. Nano range TiO_2_ powders have large specific areas and thus provide ample active sites for reaction to occur and enhance the catalytic activity. TiO_2_ nanoparticles are prone to agglomerate which results in a decrease in the photocatalytic activity. Therefore, significant efforts have been made to minimize the agglomeration of TiO_2_ nanoparticles, such as the use of supported methods, coating technology and so on^21^.

HNTs as a support can also be used for nanoparticles synthesis to avoid their agglomeration^11,22–24^. TiO_2_ with HNTs provides large specific surface area and also due to the mesoporous structure of HNTs, they may be potentially used as adsorbents, catalysts and catalyst support^25–27^. They have also been used to efficiently remove the organic pollutants by photocatalytic degradation in aqueous dispersions^28,29^. TiO_2_ is widely used as a suitable semiconductor for treating oil spills and decomposing organic pollutants present in water and air^30–33^. Lvov and co-workers have extensively worked with HNTs and examined its properties by modifying it with silver nanorods, Ru, Rh, Pt, Co, Fe and copper-Nickel alloy nanoparticles to analyse the antimicrobial activity and photocatalytic activity of modified HNTs^11,34,35^. Papoulis *et al*. used anatase form of TiO_2_ on palygorskite and halloysite surfaces to photocatalytical decompose NOx gas under visible-light and UV light irradiation^36^. Du, Y. and P. Zheng calcinated the TiO_2_-HNT composite samples at different temperature and for TiO_2_-HNT calcined at 300 °C, 81.6% MB is degraded after UV irradiation treatment for 4 h^37^.

In another research, a one-step solvothermal method was used by Wang *et al*. for the preparation of TiO_2_/HNTs samples for wastewater purification^21^. Zheng *et al*. fabricated an amylose–HNT–TiO_2_ composite for effective removal of methylene blue (MB) and persistent organic pollutant 4-nitrophenol (4-NP) under UV irradiation^38^. Peng H. *et al*. fabricated the ZnO-HNTs photocatalyst and observed that ZnO bonded HNTs showed higher photocatalytic performance toward photo-degradation of MB dye^9^. However, it is complicated to separate nano-sized photocatalyst from treated water, and the possible toxic health effects associated with it^39^, hence restricting its practical application.

In some studies, nano-sized TiO_2_ is immobilized or dispersed onto various materials like glass^40^, polymer^41,42^ and clay^43^. In our previous work, we have synthesized TiO_2_ nanoparticles on the surface of HNTs nanocomposite via the sol-gel method and blended it in different weight percentage (1–3 wt.%) of poly(vinyl chloride) (PVC) for the preparation of hybrid ultrafiltration membranes. The new hybrid membranes had improved permeation performance and enhanced antibacterial activity against *E. coli*.^44^. Damodar *et al*. also observed that almost 100% of *E. coli* is eradicated by 4% TiO_2_/PVDF membrane after 1 min of UV irradiation^45^. Wittmer *et al*. prepared cellulose based TiO_2_ photocatalyst to treat wastewater which acts as a precursor for the preparation of catalytically active membrane. However, after the regeneration of cellulose, a partial decrease in the catalytic activity is observed^46^. Zhang *et al*. studied the rejection of Direct Black 168 by using a TiO_2_/Al_2_O_3_ composite membrane under UV irradiation at different pH of the wastewater. They observed that 82% of the Direct Black 168 is degraded due to TiO_2_/Al_2_O_3_ composite membrane under UV irradiation after 300 min^47^.

In this study, the HNTs surface is functionalized by using organosilane ((3-Aminopropyl)triethoxysilane)) as a coupling agent so that the HNTs surface becomes suitable for TiO_2_ attachment. As the raw HNTs are less adhesive in nature, there is a risk of leaching out of TiO_2_ during the course of the experiment. Thus the raw HNTs functionalized with organosilane coupling agent for proper attachment between TiO_2_ and HNTs. After functionalization of HNTs, TiO_2_ nanoparticles are synthesized on the surface of HNTs (TiO_2_@HNTs) with the help of titanium (IV) isopropoxide (TIP) and ethanol solution. Novel photocatalytic PVC membranes are prepared by blending the TiO_2_@HNTs photocatalyst in two different concentration i.e. 2 wt.% and 3 wt.% using a non-solvent phase induced method. The main aim for fabricating the TiO_2_@HNTs photocatalyst and photocatalyst membrane is to exploit the photocatalytic activity of  functionalized HNTs while avoiding the aggregation of TiO_2_ nanoparticles and simultaneously reducing the electron-holes recombination rate of TiO_2_ surface, thus improving the photocatalytic activity.

The structure and morphology of TiO_2_@HNTs photocatalyst and photocatalytic PVC membrane are characterized by fourier transform infrared spectroscopy (FT-IR), X-ray diffraction (XRD), scanning electron microscopy (SEM), energy dispersive X-ray (EDX), transmission electron microscopy (TEM), UV-Vis spectrophotometer and photoluminescence (PL). The photocatalytic activity of TiO_2_@HNTs photocatalyst (TiO_2_@HNTs) alone and photocatalytic PVC membranes (TiO_2_@HNTs M2 and TiO_2_@HNTs M3) are investigated by using methylene blue (MB) and rhodamine B (RB) dyes degradation test. After MB and RB dye degradation test, end products are identified by a liquid chromatography–mass spectrometry (LC-MS).

## Result and Discussions

### Characterization of TiO_2_@HNTs and photocatalytic PVC membranes

**Figure 1:**
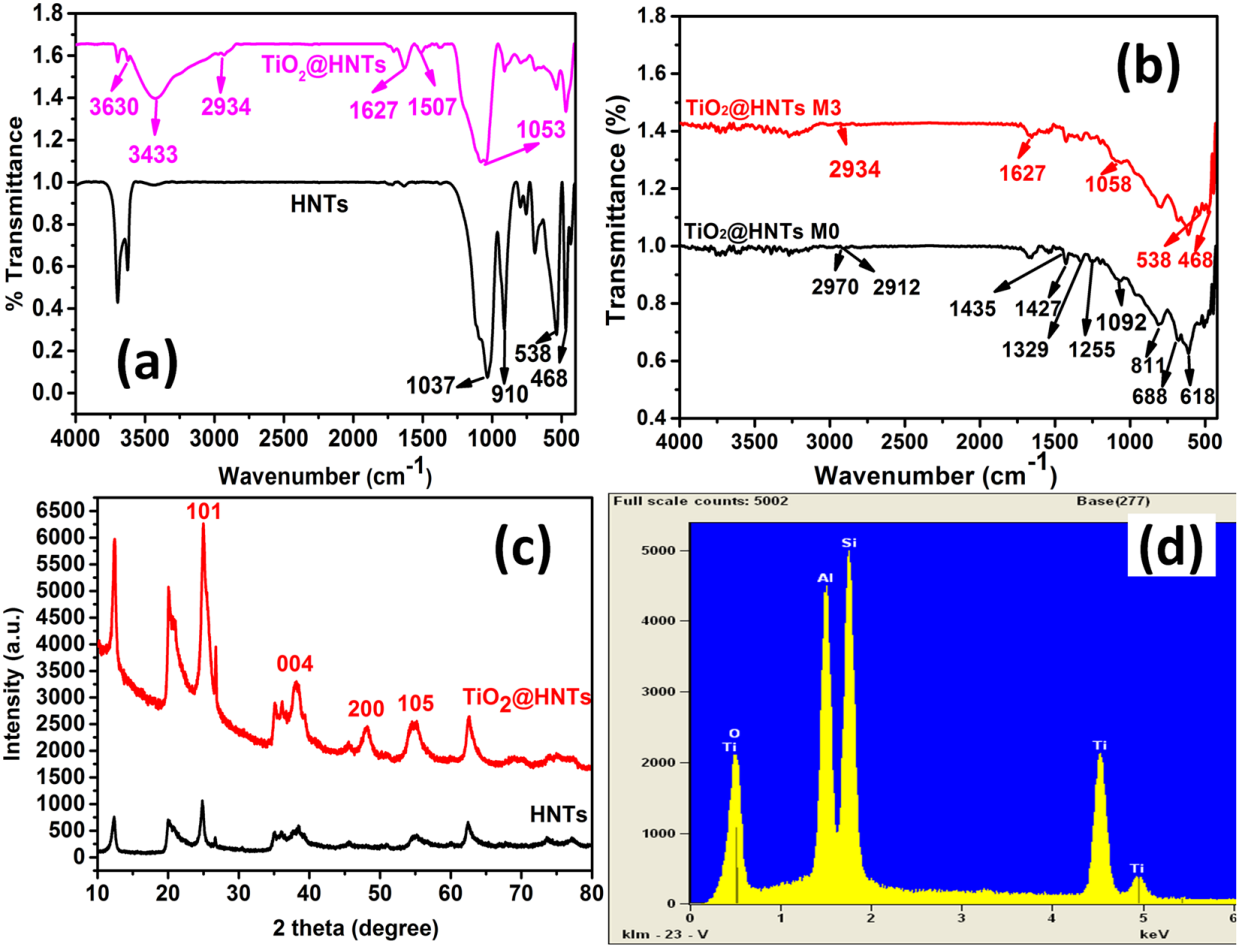
FT-IR spectra of raw HNTs and TiO_2_@HNTs (**a**), raw PVC membrane (TiO_2_@HNTs M0) and photocatalytic PVC membrane (TiO_2_@HNTs M3) (**b**), XRD peaks of HNTs and TiO_2_@HNTs (**c**) and (**d**) EDX spectra of TiO_2_@HNTs photocatalyst.

FT-IR spectra as shown in Fig. 1(a), confirmed the presence of functionalized HNTs after modification. When raw HNTs^48^ were compared with the TiO_2_@HNTs, some new FTIR peaks were observed in the spectrum like stretching CH_2_ vibration band around 2934 cm^−1^, and the deformation CH_2_ and Si-CH vibration at 1627 cm^−1^ and1507 cm^−1^ respectively_._ Apart from these peaks, broad peak of O-H stretching of water at 3433 cm^−1^ was also seen. These observations confirmed the presence of silane coupling agent^49^. Silane coupling agent is used because its main function is to ensure proper bonding between TiO_2_ and HNTs^49–52^. If this functionalization is not done, due to the absence of chemical conjugation^53^, the TiO_2_ may leach out during the course of the experiment because of the less adhesive nature of raw HNTs^54,55^. The TiO_2_@HNTs possess some significant signals, like distortions of aluminium-oxygen-silicon and silicon-oxygen-silicon bonds at 538 and 468 cm^−1^ respectively; and -OH groups of the inner hydroxyl groups at 909 cm^−1^. Furthermore, for comparison, the broad stretching band of silicon-oxygen at about 1037 cm^−1^ shifts to about 1058 cm^−1^, indicating hydrogen bonding between TiO_2_ and HNTs^21^. In Fig. 1(b), ATR-FT-IR spectra of TiO_2_@HNTs M0 and TiO_2_@HNTs M3 with the principle bands of PVC were depicted. FT-IR spectrum of PVC reflects expected distinctive absorptions: 2970–2912 cm^−1^ attributed to stretching C-H of CHCl and stretching C-H of CH_2_ group, 1435 cm^−1^ and 1427 cm^−1^ attributed to deformation wagging of CH_2_ group, and 1331–1255 cm^−1^ shows the stretching of the C-H of CHCl groups. Stretching C-C (1092 cm^−1^), rocking CH_2_ (811 cm^−1^), stretching C-Cl attributed to 688 cm^−1^ and 618 cm^−1^ respectively^56^. In Fig. 1b, the peaks of photocatalyst PVC membrane in the ATR-FT-IR results resembles the characteristic peaks belonging to both TiO_2_@HNTs M0 and TiO_2_@HNTs M3. Other characteristic absorption peaks for TiO_2_@HNTs in the spectrum of the membrane was not clearly identified because of the overlap with absorption peaks of PVC polymer or the IR beam might not be able to penetrate properly enough to get distinct peaks of HNTs^57^ in FTIR spectra (Fig. 1b).

The results of XRD clearly shows the specific peaks as shown in Fig. 1(c) which compares the XRD results of HNTs and TiO_2_@HNTs synthesized. The peaks depicted for HNT sample can be translated into the characteristic peaks of halloysite shown in Fig. 1(c). Two fresh peaks, however, can be noticed at 2θ = 48° and 54.1° and a stronger peak at 2θ = 25.3° alongside with decline in the halloysite peaks due to the sol-gel method. Based on JCPDS 21–1272, all peaks related to TiO_2_ properties can be indexed to the (101), (004), (200) and (105) planes of TiO_2_ structure^58,59^. This verifies the successful preparation of TiO_2_@HNTs. Similar results of XRD pattern also described by the Ghanbari *et al*., in their study for the fabrication of high-performance thin film nanocomposite membranes^60^. Furthermore, in the EDX spectrum (Fig. 1d) confirms the loading of TiO_2_ nanoparticles on the surface of HNTs with 24.42 wt%. Intense peaks of Ti and oxygen at 0.5 eV were observed which confirmed the presence of TiO_2_ nanoparticles on the surface of HNTs.

**Figure 2:**
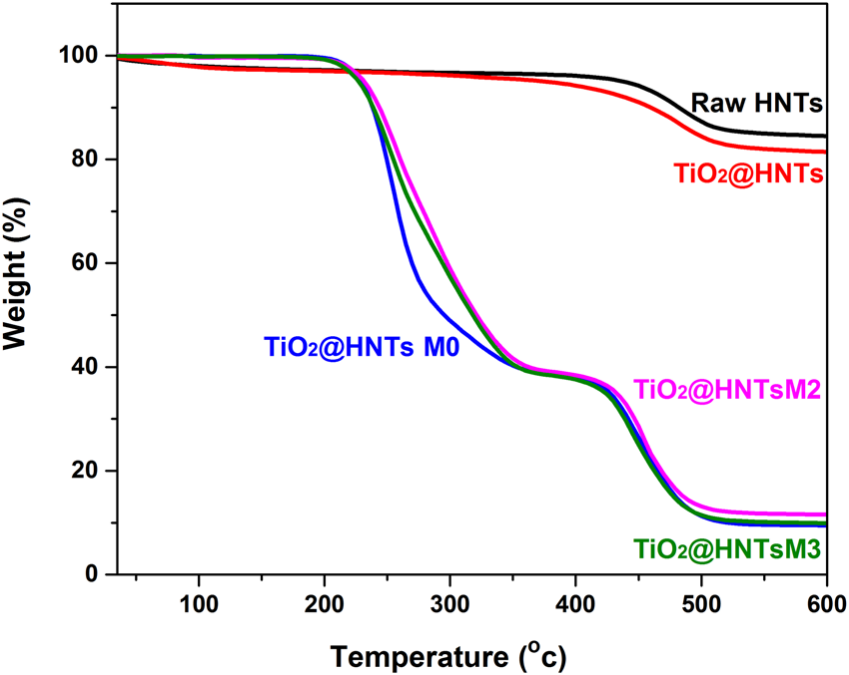
TGA curves of raw HNTs, TiO_2_@HNTs and photocatalytic PVC membrane (TiO_2_@HNTs M0- TiO_2_@HNTs M3).

Thermal behaviour of TiO_2_@HNTs and TiO_2_@HNTs/PVC blends was investigated from room temperature to 600 °C temperature range. The temperature was raised at the rate of 10 °C/min (Fig. 2). The raw HNTs showed a weight loss between 50 °C and 150 °C, which may be due to reduction in adsorbed water molecules^61^; and structural dehydroxylation of structural Al–OH groups between 450–550 °C^62^. Decomposition of the (3-Aminopropyl) triethoxysilane causes an additional weight loss in TiO_2_@HNTs between 250 °C and 425 °C when compared with the raw HNTs^63^ suggesting that the thermal stability, as well as the purity of the nanotubes, was high. PVC membrane (TiO_2_@HNTs M0–0 wt.%) and TiO_2_@HNTs membranes (TiO_2_@HNTs M2 and TiO_2_@HNTs M3) showed a different behaviour. The weight loss of the sample (around 60%) was mainly between temperature range 220 °C to 300 °C. The rest of the sample was thermally decomposed at 500 °C as shown in Fig. 2

**Figure 3:**
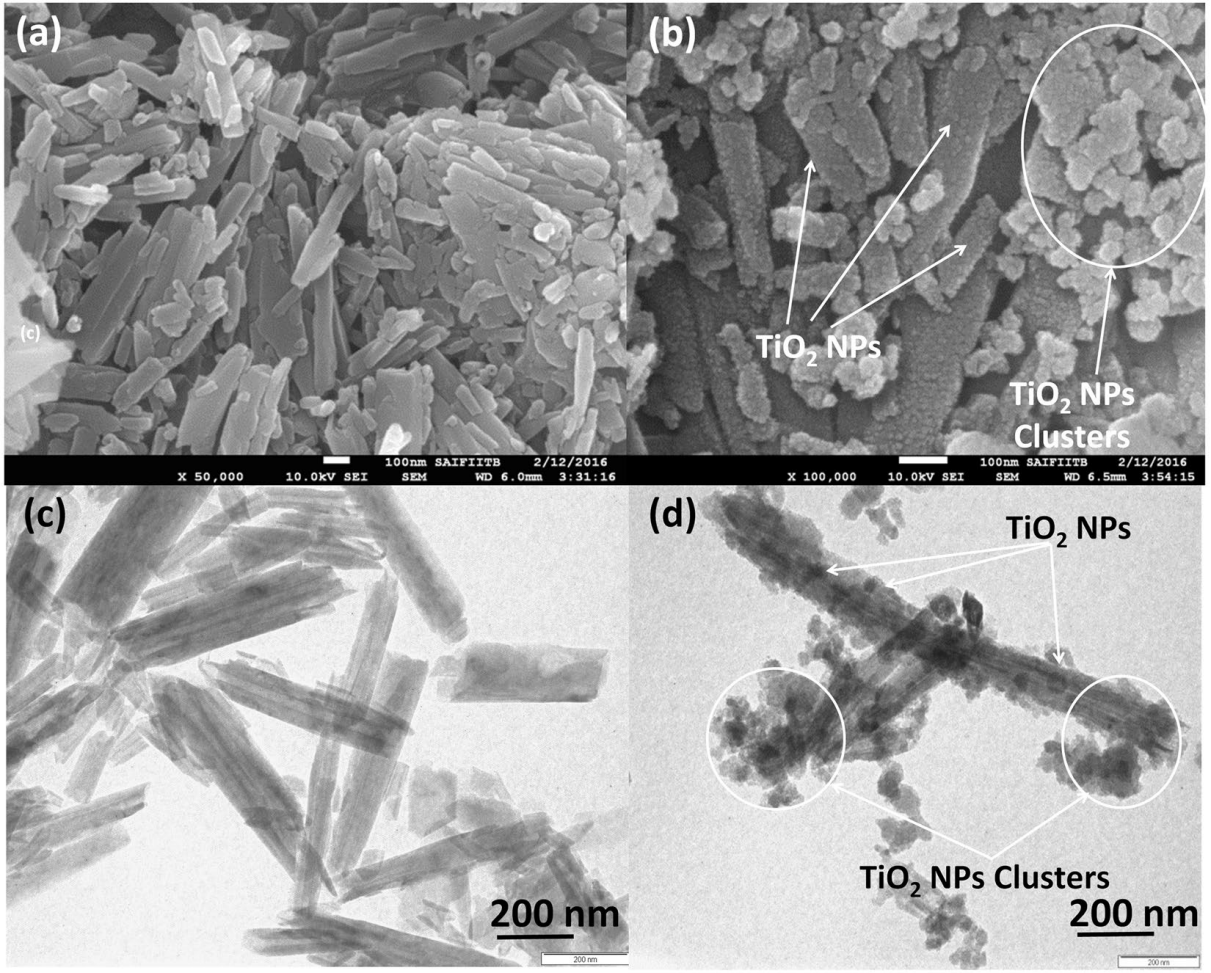
FEG-SEM of (**a**) raw HNTs, (**b**) TiO_2_@HNTs and TEM images of (**c**) raw HNTs and (**d**)TiO_2_@HNTs.

FEG-SEM and TEM were done to analyse the structural and morphological characteristics of raw HNTs and TiO_2_@HNTs photocatalyst (Fig. 3). The TEM images of raw HNTs clearly show the hollow tubular structure with a diameter of 50–70 nm and length of 0.5–2 µm (Fig. 3c). The outer surface of HNTs was made up of silica and was surrounded by multi-walled aluminol layer. In Fig. 3b,d TiO_2_ nanoparticles were seen to be randomly deposited on the surface of HNTs. Few aggregation or cluster of nanoparticles was observed in FEG-SEM and TEM analysis (Fig. 3b,d) which was formed when photocatalyst was separated from the solution during the fabrication process. The size of small particles and cluster size of this TiO_2_ range from 10 nm to 80 nm. The presence of TiO_2_ nanoparticles on the surface of HNTs confirms successful loading on HNTs.

**Figure 4:**
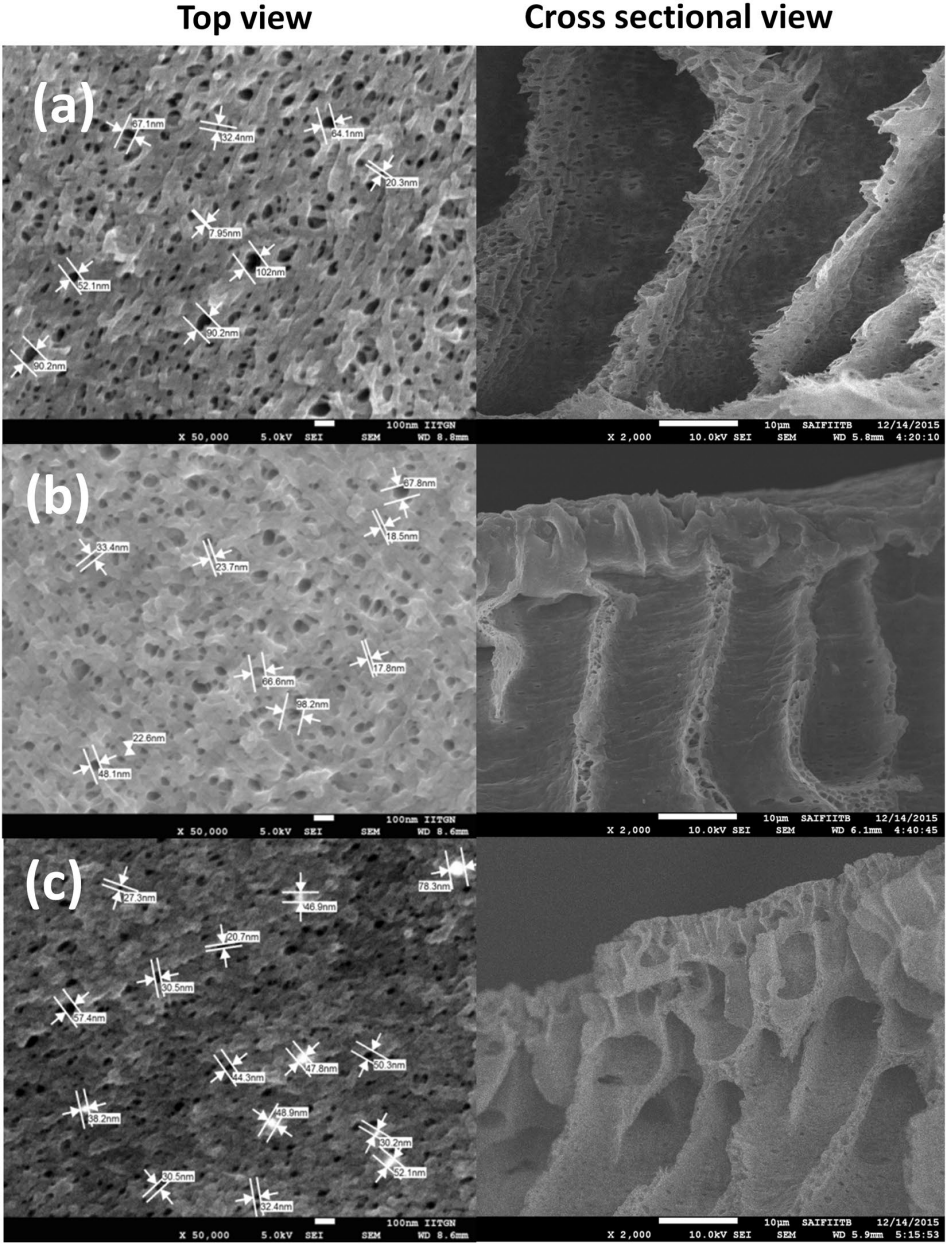
FEG-SEM images (top and cross-sectional view) of raw PVC membrane (**a**), and photocatalytic PVC membranes TiO_2_@HNTs M2 (**b**) and TiO_2_@HNTs M3 (**c**) samples.

All the membrane possessed typical asymmetric structure like that of ultrafiltration membranes and no distinct difference between the TiO_2_@HNTs M0 (Fig. 4a) and the TiO_2_@HNTs M2 and TiO_2_@HNTs M3 (Fig. 4b,c) on the top surface was observed. Only large pores were observed on the membrane surface as shown in Fig. 4(b,c) when compared with TiO_2_@HNTs M0 (Fig. 4a). FEG-SEM images shows top surface and cross-sectional view of TiO_2_@HNTs M0 and TiO_2_@HNTs M2 and TiO_2_@HNTs M3 membranes. All membrane samples were homogeneous and asymmetric in nature with macro-voids and finger-like structures formed because of the high mutual diffusivity of DMAc and water. This porous structure indicates that TiO_2_@HNTs increases the membrane porosity without altering the membrane morphological structure^64^.

**Figure 5:**
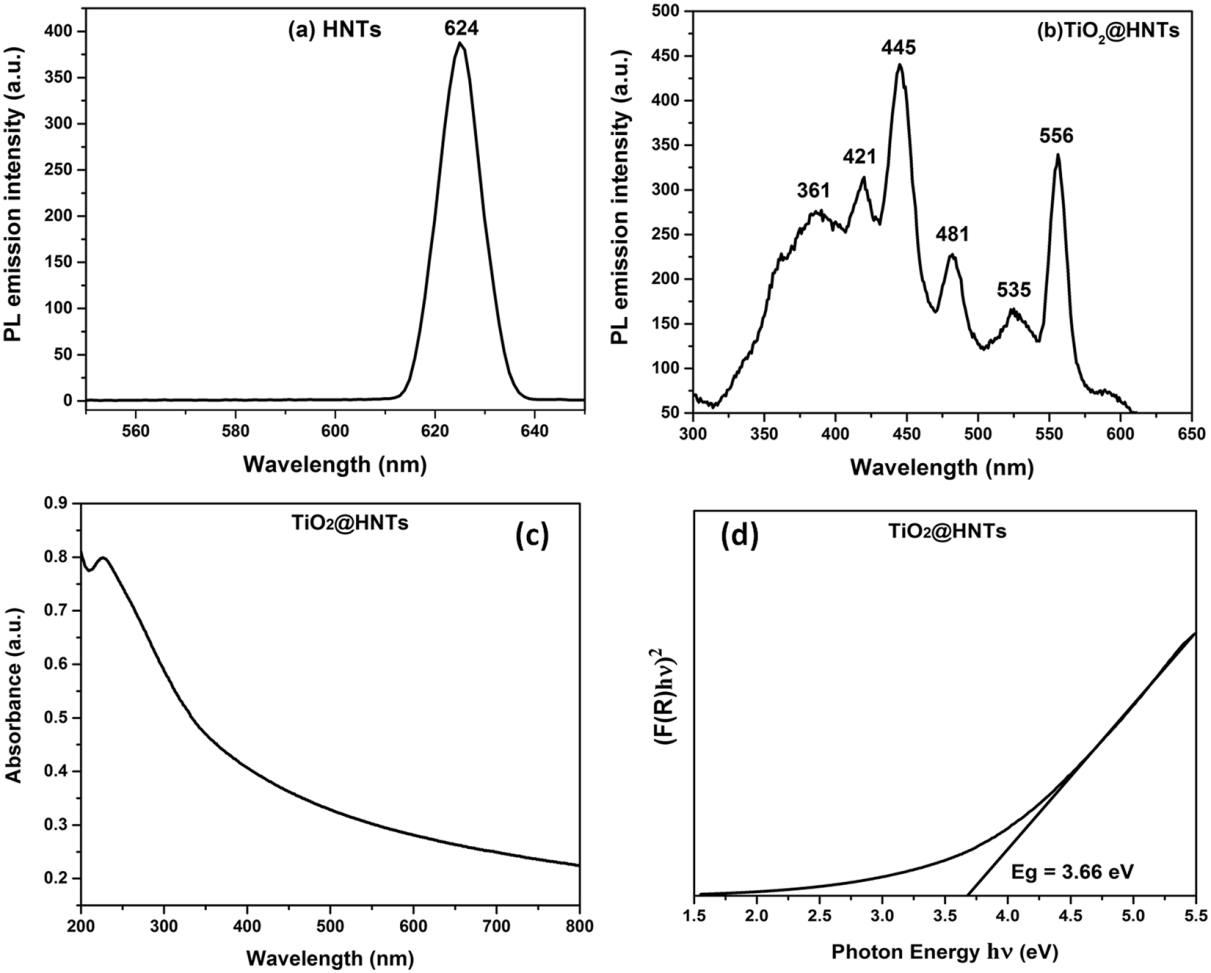
Photoluminescence (PL) emission spectra of (**a**) HNTs (**b**) TiO_2_@HNTs (**c**) UV absorption spectra of TiO_2_@HNTs and (**d**) band-gap energy spectra of TiO_2_@HNTs.

To investigate the recombination of the free carrier in HNTs, photoluminescence (PL) emission spectrum was analysed. One broad PL peak centred under excitation of 450 nm visible light irradiation was observed in Fig. 5a. The broad-band at 624 nm indicates that the light for excitation can initiate electron transition from the valence band (VB) to the conduction band (CB) in HNTs. As a result of this transition, the electron/hole pair can be generated which then further recombine radiatively to give broad and strong PL signal under 450 nm light irradiation. The PL emission spectrum of TiO_2_@HNTs was recorded at an excitation wavelength of 220 nm (high absorption region). The major peak at 361 nm (lower than the band edge emission) in PL emission spectra (Fig. 5b) was due to the band to band transition. At longer wavelengths in the visible region, the emission peaks at 421, 445, 481, 535 and 556 nm reflect the surface state emissions, located within the band gap of TiO_2_@HNTs^65^_._ The oxygen vacancies and surface hydroxyl groups present on the surface of TiO_2_ and HNTs acts as excellent traps for charge carrier, reducing the electron-hole recombination rate. Presence of oxygen vacancies from shallow trap state near the adsorption band edge act as efficient electron trap centres or colour centres. UV-Vis spectroscopy was used to study the light absorption properties. Figure 5c displays the UV-Vis absorption spectra of the prepared materials obtained. The band gap of pure TiO_2_ is 3.2 eV and the absorption edge of the TiO_2_@HNTs material is around 220 nm corresponding to the band gap energy (Eg) of 3.66 eV (Fig. 5d). This difference is due to the addition of HNTs because of which the modified photocatalyst gets excited to produce more electron-hole pairs under light irradiation, resulting in higher photocatalytic activity^66,67^.

### Photocatalytic activities of TiO_2_@HNTs photocatalyst and photocatalytic PVC membranes for the degradation of MB and RB dye solutions

**Figure 6:**
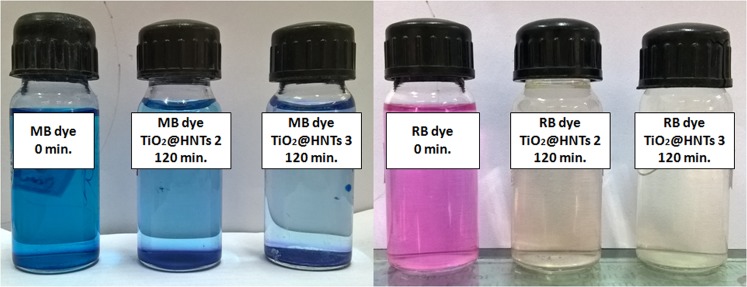
Digital photograph of MB and RB dyes under 120 min of UV irradiation.

In Fig. 6 images of MB and RB dye degradation under 120 min of UV irradiation can be seen. Initially, the colour of MB dye solution was blue and RB dye solution was pink. After 120 min, the colour of TiO_2_@HNTs 2 and 3 reaction mixture changed from blue to light blue in case of MB dye and from pink to colourless solution in case of RB dye.

**Figure 7:**
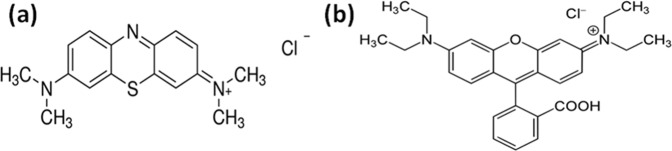
Structures of (**a**) methylene blue (MB) and (**b**) rhodamine B (RB) dyes.

**Figure 8:**
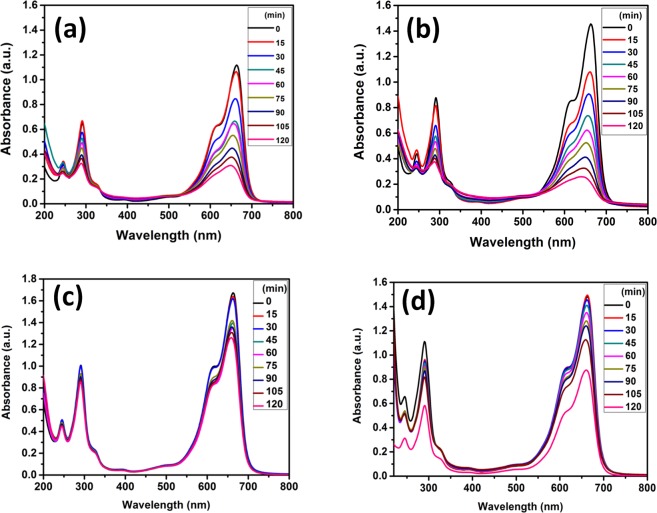
Photocatalytic degradation of MB dyes absorption spectra at different time intervals-TiO_2_@HNTs 2 & 3 (**a**,**b**), photocatalytic PVC membranes TiO_2_@HNTs M2 and TiO_2_@HNTs M3 (**c**,**d**).

**Figure 9:**
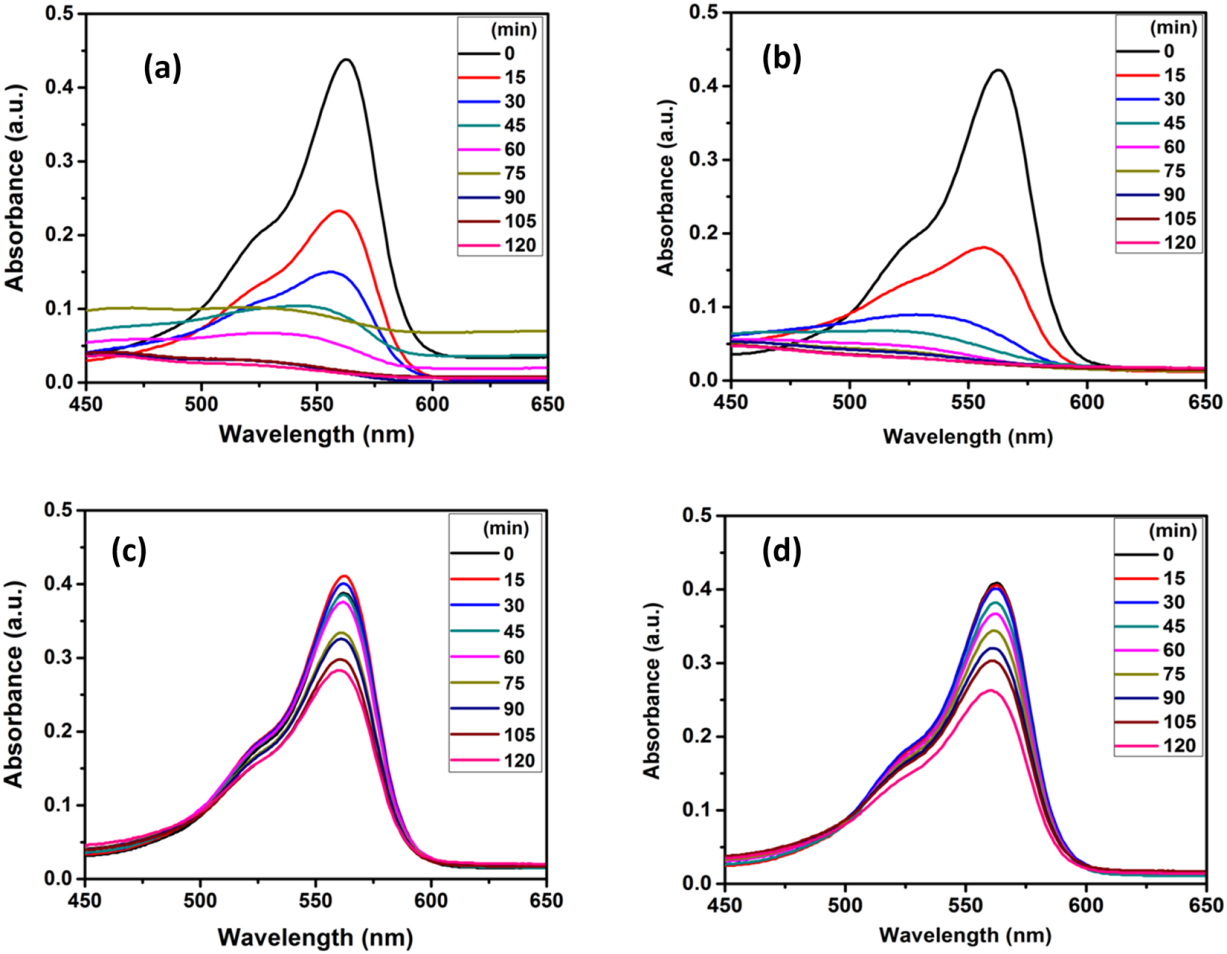
Photocatalytic degradation of RB dyes absorption spectra at different time intervals-TiO_2_@HNTs 2 & 3 (**a**,**b**), photocatalyst PVC membrane TiO_2_@HNTs M2 and TiO_2_@HNTs M3 (**c**,**d**).

This decolourization was due to degradation of dye and was highest in the case of TiO_2_@HNTs 3. The chemical structure of MB and RB dye is shown in Fig. 7(a,b). To further confirm the degradation of dye, LC-MS chromatographic separation analysis was done. For the analysis, the dye solution was collected after 120 min of UV irradiation. As seen in Fig. 8(a–d), maximum absorption of MB dye in the visible region was at 664 nm and in the UV region two peaks located at 245 and 292 nm. The highest peak of MB dye at 664 nm is due to its centre benzene ring comprising sulphur and nitrogen whereas the two dimethylamine substituted aromatic ring exhibits its peak in the UV region at 245 and 292 nm. These high peaks at 664 nm gradually diminish with time in the presence of TiO_2_@HNTs 3 under UV irradiation. Similarly, for RB dye, the characteristic absorption peak at 554 nm as shown in Fig. 9(a–d) also degrades with time. The *n* → *π* transition of C=N, C=O groups in the aromatic ring of the RB dye structure (Fig. 7b) is responsible for the colour of solution and its absorbance at 554 nm. During the reaction process, when the dye structure was disrupted by the TiO_2_@HNTs 3 photocatalyst, the absorption intensity of RB dye decreased rapidly with change in colour from pink to a colourless solution.

The shifts in the absorption spectra of both the dye solutions were studied during photocatalytic degradation analysis (Figs 8 and 9). The absorption peak of MB dye solution was at 664 nm which decreased gradually due to dye degradation and attained its lowest value at 120 min (Fig. 8a,b). The reason for the decrease in absorption peaks was attributed to the fact that TiO_2_@HNTs photocatalyst cleaves the aromatic ring of the dye molecules and initiate its degradation^68^. In the case of RB dye solution, highest and characteristic peak was seen at 562 nm, which dipped quickly in the first 15 min of degradation and was lowest after 120 min (Fig. 9a,b). The MB dye was degraded up to 83.21% and 87.47% (Fig. 8a,b) for TiO_2_@HNTs 2 and TiO_2_@HNTs 3 respectively. In the case of RB dye, 2 wt.% of photocatalyst was sufficient enough for degradation of 20 mg/L of RB dye and the degradation rate for both the 2 wt.% and 3 wt.% photocatalyst was 96.84% and 96.87% (Fig. 9a,b) respectively. Blue Shift in absorption peaks (λ max) was observed gradually with time which finally resulted in the respective lowest absorption peak of dye. The adsorption capacity of prepared TiO_2_@HNTs was also tested with MB and RB dye in dark condition at room temperature with 20 mg/L dye solution. The maximum dye adsorption efficiency of MB dye and RB dye were 17.5 mg/g and 4.8 mg/g for TiO_2_@HNTs 2 and 12.10 mg/g and 3.67 mg/g for TiO_2_@HNTs 3 respectively after 2 h. In the control experiment (HNTs 2 and HNTs 3), the removal of the MB dye was around 39.22% and 47.82% and RB dye was around 17.65% and 25.2% respectively after 2 h of irradiation. Also, there was a minor reduction in UV spectra even when TiO_2_@HNTs photocatalyst was added and both MB and RB dye solution was kept in dark. Thus it was confirmed that the degradation was due to TiO_2_ nanoparticles in the presence of UV irradiation.

Though photocatalytic PVC membranes TiO_2_@HNTs M2 and TiO_2_@HNTs M3 both exhibited photocatalytic activity (as shown in Figs 8c,d and 9c,d for MB and RB dyes respectively), but TiO_2_@HNTs M3 membrane possessed more catalytic activity as compared to TiO_2_@HNTs M2 because with the increase in the TiO_2_@HNTs concentration the catalytic activity also enhances. However, it was observed that photocatalytic activity reduced in photocatalytic PVC membranes when compared with the same wt% of TiO_2_@HNTs alone. The MB dye was degraded up to 27.19%, 42.37% (Fig. 8c,d) and RB upto 30.78%, 32.76% (Fig. 9c,d) for TiO_2_@HNTs M2 and TiO_2_@HNTs M3 respectively. The reason behind this slow rate of degradation in both TiO2@HNTs M2 and TiO2@HNTs M3 can be due to the reduction of active sites of TiO_2_@HNTs photocatalyst during membrane preparation. During phase separation process, the increase in the amount of TiO_2_@HNTs photocatalyst increases its catalytic activity but its increase after 3 wt.% in membrane casting solution may result in photocatalyst agglomeration^69^. This agglomeration hampers the number of free catalytic sites for dye degradation and also delays the phase separation process during membrane preparation^46^. When the concentration of photocatalyst increases in the solution, the turbidity of the solution increases and light is scattered more due to which screening effect occurs hampering the specific activity of the catalyst and reducing the degradation rate^70–72^. Thus an optimal amount of photocatalyst must be used for photocatalytic degradation process for increased degradation and reduced inaccuracy.

The kinetics of photocatalytic reactions can be described using the first-order reaction for concentrations (20 mg/L) of MB and RB dye solutions. The rate constants (k) and the correlation coefficient (R^2^) has been evaluated using linear regression curve of ln(C_0_/C) versus UV light irradiation time. First-order rate equations are as follows^73^:

1 $$\mathrm{ln}\,\frac{[{C}_{0}]}{[C]}={\rm{kt}}$$

Here, k is the first order rate constant (min^−1^); [C] and [C_0_] final and initial dye concentration in (mg/L), respectively.

The degradation efficiency has been calculated using^74^:

2 $${\rm{Degradation}}( \% )=(\frac{{C}_{0}-C}{{C}_{0}})\times 100$$

where C_0_ is the initial concentration of the dye and C is the concentration of the dyes after UV irradiation in the selected time interval.

**Figure 10:**
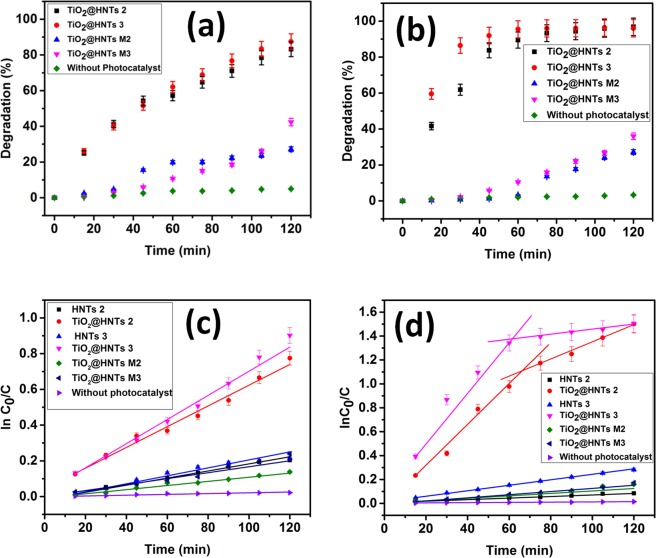
The percent rate of degradation of MB dye (**a**) and RB dye (**b**) and rate constant k for MB dye (**c**) and RB dye (**d**) for all samples.

The correlation coefficient (R^2^) was calculated to be nearly as high as R^2^ ≈ 0.95–0.99 which reiterate the suitability of the first-order reaction listed in Table 1. MB and RB degradation rate are shown in Fig. 10c,d, where the TiO_2_@HNTs photocatalyst has notably improved the photocatalytic activity. During photocatalysis of RB dye by TiO_2_@HNTs 2 and 3, dye adsorption process occurred in two steps shown in Fig. 10d. At first stage, RB molecules diffuse from the aqueous solution to the external surface of TiO_2_@HNTs or the boundary layer diffusion of RB molecules. Secondly, gradual adsorption occurred until equilibrium was reached. The linear portion of the first stage did not pass through the origin, indicating the existence of a boundary layer resistance between TiO_2_@HNTs photocatalyst and dye solution^75^ Similar observation is reported in literature^73^. Furthermore, the detailed photocatalytic mechanism is described in detail in a later section.

**Table 1:** Rate parameter of photo-catalytic activity of MB and RB dye.

Samples	K [min^−1^]	R^2^
**Methylene Blue**		
HNTs 2	0.00182 ± 0.00006	**0.990**
TiO_2_@HNTs 2	0.00605 ± 0.0002	**0.986**
TiO_2_@HNTs M2	0.00117 ± 0.00005	**0.981**
HNTs 3	0.00197 ± 0.0001	**0.968**
TiO_2_@HNTs 3	0.0073 ± 0.0002	**0.993**
TiO_2_@HNTs M3	0.00193 ± 0.0001	**0.969**
**Rhodamine B**		
HNTs 2	0.00701 ± 0.00002	**0.989**
TiO_2_@HNTs 2	**1**^**st**^ 0.01675 ± 0.001 **2**^**nd**^0.00745 ± 0.0005	**0.984** **0.983**
TiO_2_@HNTs M2	0.00134 ± 0.00009	**0.959**
HNTs 3	0.00231 ± 0.0005	**0.996**
TiO_2_@HNTs 3	**1**^**st**^0.02258 ± 0.001 **2**^**nd**^0.00236 ± 0.0002	**0.970** **0.964**
TiO_2_@HNTs M3	0.00136 ± 0.00007	**0.976**

**Figure 11:**
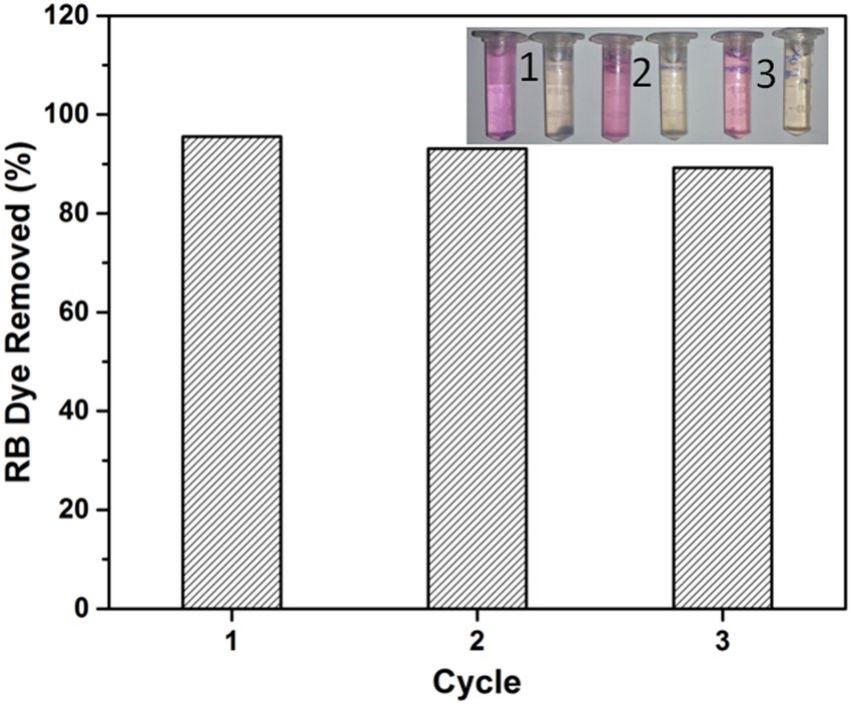
Photocatalytic stability test of TiO_2_@HNTs photocatalyst.

The stability of the TiO_2_@HNTs photocatalyst was estimated by recycling the photocatalyst for degradation of RB dye under UV light irradiation for three times. The loss of the photocatalytic activity was negligible (approximately 6% after 3 repeated runs) (Fig. 11) which signifies the stability of TiO_2_@HNTs photocatalyst in terms of its non photo-corrosive nature during the photocatalytic degradation of the model dye molecules, which proved to be very important for its practical applications.

### Photocatalytic PVC membrane activity Test with MB dye

**Figure 12:**
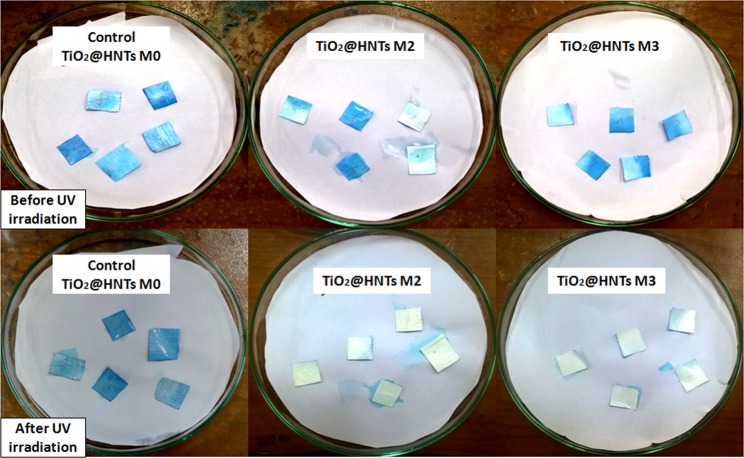
Photograph of photocatalytic PVC membrane TiO_2_@HNTs M2 & TiO_2_@HNTs M3 treated the sample with MB before and after 1 h of UV irradiation.

To evaluate the photocatalytic property of the membrane surface, control (raw PVC membrane TiO_2_@HNTs M0), TiO_2_@HNTs M2 and TiO_2_@HNTs M3 samples were treated with UV light without stirring for 1 h (Fig. 12). After 1 h of irradiation TiO_2_@HNTs M2 and TiO_2_@HNTs M3, sample colour disappears from the membrane surface. On the contrary, the change in colour in control experiment was less after irradiation of UV light. The change in colour of membrane of the control sample was seen to be least when irradiated with UV light, which was contrary to the result of samples of TiO_2_@HNTs M2 and TiO_2_@HNTs M3. TiO_2_ acts as a semiconductor and presence of UV light results in the formation of electrons and holes. These photo-generated electrons hence formed, reduces Ti (IV) cations to the Ti (III) state and the holes oxidise O_2_^−^* anions^76^. Simultaneously O_2_ atoms are propelled out producing a set of O_2_ vacancies on the surface. These vacancies were filled by water molecules present in the environment and adsorbed OH groups are formed on the surface, increasing the hydrophilicity of the surface^77^. Also, the radicals hence produced can degrade the dye molecules present around the membrane surface (detail mechanism explained in the mechanism section). For the reproducibility, a test procedure was repeated with five membrane pieces for each sample. MB dye showed better contrast while taking digital images and hence was preferred for photographic images.

### Photocatalytic Mechanism of TiO_2_@HNTs photocatalyst and Photocatalytic membrane

**Figure 13:**
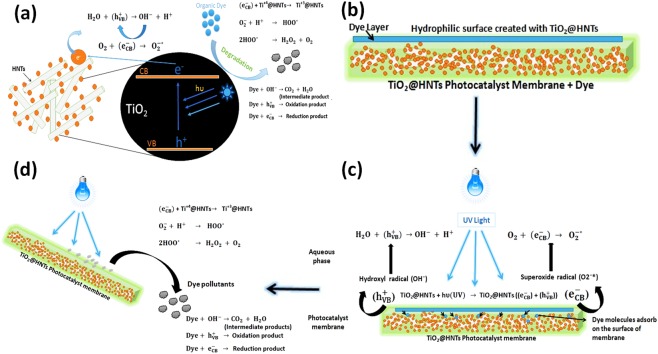
Schematic representation of the photocatalytic activity of TiO_2_@HNTs photocatalyst (**a**) and photocatalytic membrane (**b** to **d**).

When irradiated with UV light, the HNTs do not get excited, rather it acts as an electrical insulator and hence any charge generated on TiO_2_ surface during UV irradiation cannot be transferred to HNTs. These electrostatic attraction and repulsion forces contribute together for an efficient movement and separation of e^−^ and h^+^ on TiO_2_^1^. Apart from being a charge carrier separator, HNTs also enhance dye degradation. Due to the negatively charged surface of HNTs, the dye molecule (cationic in nature) are brought closer to the TiO_2_, increasing the adsorption rate of the dye molecules. The detailed mechanism of the photocatalyst TiO_2_@HNTs and photocatalyst membrane are shown in Fig. 13. When TiO_2_@HNTs is irradiated by UV light, a photoelectron moves from valence band of TiO_2_@HNTs to the empty conduction band. This photon has the energy (hυ) equal to or greater than the band gap. Thus a hole is created in VB (${{\rm{h}}}_{{\rm{VB}}}^{+}$) and an electron (${{\rm{e}}}_{{\rm{CB}}}^{-}$) in CB generated as shown in Fig. 13(a).These (${{\rm{h}}}_{{\rm{VB}}}^{+}$) then produce OH* radical after reacting with H_2_O. Now, these OH* radicals act as a potent oxidising agent and oxidise adsorbed organic molecules which are in near vicinity of TiO_2_@HNTs surface Fig. 13b. Simultaneously O_2_ atoms are propelled out producing a set of O_2_ vacancies on the surface. These vacancies are filled by water molecules present in the environment and results in the formation of adsorbed OH groups on the surface, increasing surface hydrophilicity^77^. These photo-generated electrons hence formed, reduces Ti^+4^@HNTs cations to the Ti^+3^@HNTs state and the holes oxidise O_2_^−^* anions. Also, the radicals hence produced can degrade the dye molecules present around the membrane surface as shown in Fig. 13c. The efficiency of the breakdown of these organic molecules depends upon their stability and structure. OH* radicals also degrade the pollutant present around it. The electrons in (${{\rm{e}}}_{{\rm{CB}}}^{-}$) conduction band meanwhile reacts with O_2_ and generate superoxide radicals (O_2_^−^*), which accelerates oxidation process (Fig. 13d) and also hinders any further e−/hole recombination formation, thus maintaining electron neutrality within the TiO_2_ molecule. The H^+^ formed in reaction further reacts with O_2_^−^* formed and protonates the hydroperoxyl radical (HO_2_^−^*). Hence finally hydroxyl radical (OH^*^) is formed, which is highly reactive in nature.

To understand the role and the involvement of active species in degradation process, control experiments were performed using scavengers for the photo-generated holes and free radicals. Photo-degradation of MB and RB dyes were investigated in the presence of UV light with the TiO_2_@HNTs photocatalyst to observe the role and the importance of degradation by free radicals. Isopropanol (IPA) and Tert-butyl alcohol (TBA) were used as H^+^ and hydroxyl radical (OH*) scavenger respectively. In both reactions with MB and RB dye, H^+^ showed no significant effect on photocatalytic degradation, while OH* free radical affected the MB and RB photocatalytic degradation reactions. Adding TBA (0.02 mmol) in the reaction, photocatalytic degradation decreased from 87.47% to 44% in case of MB dye while in case of RB dye degradation percentage decreases from 96.87% to 72%. This proved that OH* free radicals were generated during photodegradation of dye. Several researchers^78^ have suggested that the OH* radical produced by the oxidation of water or OH^−^ radicals by holes at the surface, diffuses towards the solution to oxidise the organic compound. H_2_O_2_ and hydroxylated degradation products were formed during the reaction and the efficiency of degradation increases significantly when H_2_O_2_ is formed in the presence of UV radiation. This was because of free hydroxyl radicals (which act as powerful oxidizing agent) generated by the dissociation of H_2_O_2_ in the presence of UV irradiations. Moreover, a high concentration of hydroxyl peroxides itself acts as a scavenger which reduces the concentration of hydroxyl radicals and compound elimination efficiency. The generated hydroxyl radicals attack the MB and RB dye structure at different sites like un-saturation points etc. In several such attacks, the MB and RB dyes get converted into CO_2_ and hetero-atoms which are further mineralized as mentioned in Supplementary File Tables S2 and S3. The combination of TiO_2_@HNTs photocatalyst has both the advantages of being an efficient charge carrier separator and good absorbent for positively charged molecules^1^. The photocatalytic activity was also enhanced due to the absorptivity of HNTs and the crystalline TiO_2_ nanoparticles which facilitate the interaction of dye and reactive TiO_2_@HNTs photocatalyst. Also, the agglomeration of TiO_2_ nanoparticles was avoided by the homogeneous dispersion of TiO_2_ nanoparticles on the HNTs surface.

The experimental data confirm that after 120 min of UV irradiation, the dye solution gets degraded with the formation of intermediate and end products. A mass spectroscopic (MS) study of the dye solution was also done to determine the intermediate and end products which were formed due to the cleavage of aromatic rings during the dye degradation process and eluted out at different retention time as per their mass and suggested structures. The difference in the concentration and composition of the products lead to many peaks with different intensities. Mass spectra and the possible structures of the dye degradation products are listed in Supplementary Table S2 and Table S3 for MB and RB dye^79–81^. The demethylation cleavage has also been reported in the literature during the photocatalytic degradation^79,80,82–84^.

For evaluation of the activity of a photocatalyst, commonly time dependence of the concentration loss of dye under UV irradiation is measured. However, there are many factors that govern the reaction rate and kinetics. These experimental conditions include the concentration of the photocatalyst, the surface area of the photocatalyst, the amount of the photocatalyst used in the experiment and the UV light intensity and more. Table 2 summarises some recently synthesized photocatalyst with their photocatalytic experimental data.

**Table 2:** Studies of photocatalyst and photocatalyst membrane performance as reported in the literature.

S.No.	Photocatalyst/Photocatalytic membrane	Method	Target	Performance	Time	Ref.
1	TiO_2_@HNTs	Sol-gel method Phase inversion	RB and MB	TiO_2_@HNTs degraded up to 87%, 96% and PVC photocatalytic membranes degraded up to 42.37%, 32.76% MB and RB respectively	2 h	**Present study**
2	TiO_2_@HNTs	Sol-gel method	MB	MB dye degradation upto 81.6%	4 h	^37^
3	TiO_2_@HNTs	Solvothermal method	Acetic acid Methanol	Degradation of acetic acid 3488.63 μmol/g and methanol 729.37 μmol/g	1–2 h	^21^
4	TiO_2_ nanotube membranes	Through-hole morphology	RB	RB dye degradation up to 28%	5.3 h	^89^
5	TiO_2_-Polyvinylidene fluoride	Plasma-induced graft polymerization	Reactive Black 5 dye	Removed 30–42% of 50 mg/L aqueous Reactive Black 5 dye	—	^90^
6	TiO_2_-Al_2_O_3_ membrane	Sol-gel processing method	Methyl orange	Removed 27% of 5 mg/L aqueous methyl orange	9 h	^91^

## Conclusion

Utilization of naturally present HNTs as photocatalyst support is advantageous for the synthesis of TiO_2_@HNTs photocatalyst nanoparticles due to its size and shape dependent photocatalytic properties. In this study, TiO_2_@HNTs photocatalyst and photocatalytic PVC membranes are  synthesized. The prepared photocatalyst is stable and exhibits enhanced photocatalytic activity for the degradation of MB and RB dye solution under UV irradiation. The photocatalytic PVC membrane also exhibits similar photocatalytic activity against MB and RB dye but the degradation is slower as compared to the TiO_2_@HNTs photocatalyst of the same weight.

Due to the electrostatic interaction between TiO_2_ and HNTs surface, the photocatalyst has more e^−^ and h^+^ pairs resulting in high photocatalytic activity. In the case of MB and RB dye which are positively charged, HNTs improved the supply and stability of the photo-generated charges and enhanced the absorption capability of the dye molecule on the photocatalyst. This was because of electrostatic attractive and repulsion forces originating from the negatively charged HNTs surface. The stability of the TiO_2_@HNTs photocatalyst is non photo-corrosive nature during the three consecutive cycle of photocatalytic degradation of the model dye molecules, which is very important for its practical applications. The MB and RB degradation catalysed by a TiO_2_@HNTs and photocatalyst PVC membrane followed the first-order kinetic model. Therefore, the capabilities of nano range TiO_2_@HNTs photocatalyst to degrade dyes may be exploited for wastewater purification in various textile and chemical industries.

## Materials and Methods

### Materials

HNTs, Titanium (IV) isopropoxide(TTIP) and Poly (vinyl chloride) polymer were furnished by Sigma-Aldrich. (3-Aminopropyl)triethoxysilane (silane coupling agent) was purchased from Himedia. Tert-butyl alcohol (TBA) (99%) and isopropanol (IPA) (99%) were purchased from s-d Fine Chem. Ltd, Mumbai. Rhodamine B (RB) and Methylene blue (MB) dyes were provided by Colourtex Pvt. Ltd., Surat, Gujarat. All other chemicals were also of analytical grade and were used without any purification. The water used was Elix millipore pure water (DI).

#### TiO_2_@HNTs photocatalyst preparation

For the preparation of photocatalyst, moisture was removed from the inner/outer surfaces of raw HNTs by drying them for 4 h at 400 °C. 30 ml of silane coupling agent was mixed with 100 ml of toluene and 10 g of dried HNTs was then added into the silane-toluene solution to make it functional^53^. The mixture was then stirred at 125 °C for 18 h. After this, the mixture of functionalized HNTs was centrifuged and then washed with isopropanol (3–4 times). Vacuum drying chamber was further used for drying of the pellet at 60 °C. To synthesize TiO_2_ nanoparticles on functionalized HNTs surface, 1 g of silane HNTs was mixed with titanium (IV) isopropoxide (TIP) - ethanol solution (ratio 1:15) by dispersing it into the deionised water (pH value adjusted by adding HNO_3_ or NH_4_OH). This solution was then vigorously stirred for sol-gel preparation. Hydrolysis reaction was initiated when TIP solution interacted with water molecules making the solution turbid and resulting in the increase of temperature to 60–70 °C for 18–20 h. When the peptization process was complete, stirring was stopped and centrifugation was done to retrieve TiO_2_@HNTs mixture, which was then subjected to vacuum drying chamber overnight at 65 °C^85,86^. At last, the calcination of TiO_2_@HNTs was done by heating it in a muffle furnace for 2 h (400 °C)^44^.

#### Photocatalytic PVC membranes

The photocatalytic PVC membranes were prepared based on the principle of classical phase inversion method. The mixture containing TiO_2_@HNTs photocatalyst (2 wt.% and 3 wt.% by weight of PVC) and DMAc solvent (85 wt.% by weight of the solution) was stirred at 600 rpm for 1 h to get the photocatalyst dispersed properly in the solvent. For the formation of pores, PVP (1 wt.% by weight of the solution) and PVC (14 wt.% by weight of the solution) polymer were added into the mixture (shown in Table 3). For uniform dispersion, the mixture was vigorously stirred for 12 h. After stirring, a homogeneous casting solution thus obtained was then degassed (room temperature) and poured on a glass slide with the help of membrane applicator (thickness 150 µm). The glass plate was then dipped immediately in a pure water bath for 12 h (room temperature) for the proper phase inversion process. Two photocatalytic PVC membranes (Table S4) were prepared based on the weight% of TiO_2_@HNTs photocatalyst added in the membrane casting solution.

**Table 3:** Photocatalyst amount and composition of photocatalytic PVC membrane (for 20 ml) casting solution.

S.No.	Photocatalyst/photocatalytic membrane (with abbreviation)	Photocatalyst (g)	PVC (g)	PVP (g)	DMAc (g)
1.	TiO_2_@HNTs 2 (2 wt%)	0.056	—	—	—
2.	TiO_2_@HNTs 3 (3 wt%)	0.084	—	—	—
3.	TiO_2_@HNTs M0 (0 wt%)	0.00	2.80	0.2	17
4.	TiO_2_@HNTs M2 (2 wt%)	0.056	2.74	0.2	17
5.	TiO_2_@HNTs M3 (3 wt%)	0.084	2.71	0.2	17

### Characterizations

#### Fourier transform-infrared (FT-IR) spectroscopy

3000 Hyperion Microscope with Vertex 80 FTIR System (Bruker, Germany) was used for the characterization of raw HNTs, TiO_2_@HNTs and photocatalytic membrane. Scan range was 450–4500 cm^−1^.

#### Thermo-gravimetric analysis (TGA)

Samples were heated from room temperature to 600 °C at the rate of 10 °C min^−1^ under flowing nitrogen using a Diamond TG/DTA (Perkin Elmer, USA) instrument.

#### X-ray diffraction (XRD)

HNTs and TiO_2_@HNTs Photocatalyst powder samples were put into the sample collector for X-ray diffraction analysis with PANalytical, The Netherlands, scan rate 2 degrees/min. XRD peaks were recorded in the reflection mode in the angular range of 10–80° with (2 theta) angle.

#### Transmission electron microscopy (TEM)

The morphological characteristics of raw HNT and TiO_2_@HNTs were studied by a CM 200 transmission electron microscope (Philips). The samples were dispersed in deionized water, and then the suspended particles were transferred to a copper grid.

#### Field emission gun-scanning electron microscopes (FEG-SEM) with EDS

JSM-7600F FEG-SEM was used for determination of the structure of raw HNT and TiO_2_@HNTs which had an energy dispersive X-ray spectrum (EDS, Inca Energy-200) at an accelerating voltage of 200 kV.

#### Photoluminescence spectra

At room temperature, the photoluminescence spectra were recorded with a Cary Eclipse fluorospectrometer using 220 and 450 nm Ar^+^ laser as excitation source.

#### Band gap energy

For determining the absorption coefficient, optical energy gap (Eg) and nature of transitions involved, the optical absorbance spectra of TiO_2_@HNTs (at room temperature) were studied. The thickness of the quartz cuvette (t), the optical absorption coefficient (α) was determined from the measurement of wavelength (λ). Generally,the absorption coefficient (α) was related to photon energy (hν) by known equation^87,88^:

$$({\rm{\alpha }}{\rm{h}}{\rm{\nu }})={\rm{\beta }}({\rm{h}}{\rm{\nu }}-{{\rm{E}}}_{{\rm{g}}}{)}^{{\rm{n}}}$$

where, β signifies a constant known as band tailing parameter, Eg: energy of the optical band gap and n: power of the transition.

To convert the absorption spectra, in place of α, the kubelka-Munk function was used to eliminate any tailing contribution from UV spectra. The following function was applied to convert the absorption spectra:

$$F(R)=\frac{{(1-R)}^{2}}{2R}$$

where *R*, the reflectance E_g_ values were estimated from plot of (F(*R*) hν)^2^ *versus* energy by extrapolating the linear part.

#### Liquid chromatography–mass spectrometry (LC-MS)

The chromatographic experiments with LCMS system were carried out on an Agilent 1290 Infinity UHPLC System, 1260 infinity Nano HPLC with Chipcube, 6550 iFunnel Q-TOFs (Agilent Technologies, USA) with a Column, binary pump and an autosampler. Acetonitrile was used as mobile phase solvent. The mass spectrometer was equipped with an electrospray ionization (ESI) source. The mass range was from 50 to 1000 *m/z*. Degradation products were monitored by LC-MS. Measurement conditions are listed in Supplementary Table S1.

### Methods

#### Photo-catalytic reaction experiments

The possible photocatalytic activities of the TiO_2_@HNTs 2, TiO_2_@HNTs 3, TiO_2_@HNTs M2 and TiO_2_@HNTs M3 membranes were examined by assessing the MB and RB dye degradation which was prepared in an aqueous medium. UV batch reactor was used to carry out the photocatalytic reaction at a low pressure of 125 W UV lamp (254 nm) and Photon flux (Φ) = 1.69 × 10^20^ s^−1^ m^−2^ with continuous stirring. The photoreactor was initially filled with 100 mL of a 20 mg/L aqueous dye solution along with different weight percentage of a photocatalyst for the process and also with a different weight percentage of photocatalytic PVC membranes. The different concentration and combination used in the reactorare mentioned below. A UV−visible spectrophotometer (HACH, DR 6000, USA) was used to record the absorption magnitude of the dye solution regularly and similar steps was performed for both the dyes, after regular time intervals, at wavelengths of 664 nm for MB and 562 nm for RB dye respectively. Similarly, the photocatalytic efficiency of photocatalytic PVC membranes (TiO_2_@HNTs M2 and TiO_2_@HNTs M3) were also assessed. Initially, the membranes were dried and cut into small pieces and then dispersed into the aqueous dye solution for 1 h under constant stirring, after which dye solution irradiated with UV light, the concentration was analysed by a UV−visible spectrophotometer at regular intervals.

For comparative analysis, two different weight percentage of TiO_2_@HNTs photocatalyst (accordingly photocatalyst added in membrane casting solution) and photocatalytic PVC membranes were used for this study under following conditions –

(i) MB and RB dye solution exposed to UV light in the absence of HNTs, TiO_2_@HNTs photocatalyst and photocatalytic PVC membrane (adsorption of dyes).
(ii) MB and RB dye solution irradiated with UV light with only HNTs. (denoted as HNTs 2 and HNTs 3 i.e 2 wt.% and 3 wt.%)
(iii) MB and RB dye solution were irradiated with UV light with only TiO_2_@HNTs photocatalyst (denoted as TiO_2_@HNTs 2 and TiO_2_@HNTs 3, a similar weight of photocatalyst added in membrane casting solution). In addition, we have also taken a higher concentration of photocatalyst in the reaction.
(iv) MB and RB dye solution irradiated with UV light with photocatalytic PVC membranes (denoted as TiO_2_@HNTs M2 and TiO_2_@HNTs M3 i.e 2 wt.% and 3 wt.% photocatalyst added in membrane casting solution as mentioned in membrane preparation section)
(v) Scavenger effects on the MB and RB dye degradation reaction were also tested by using TBA and IPA (with 0.2 mmol solutions) as hydroxyl radical (OH^*^) and H^+^ scavengers respectively.

#### Batch mode adsorption experiments

Adsorption experiments were performed containing 2 wt.% and 3 wt.% of TiO_2_@HNTs photocatalyst and dye solution (20 mg/L MB and RB). The conical flasks were placed on a magnetic stirrer for 2 h. MB and RB dye concentrations in the solution were analyzed by UV-Vis spectrophotometer at different time intervals during the reaction. The amount of dye on TiO_2_@HNTs photocatalyst adsorb was calculated from the following equation

$${q}_{e}=\frac{({c}_{i}-{c}_{f})\times V}{M}$$

where, q_e_ (mg/g): the amount of dye adsorbed, C_i_ and C_f_ (mg/L): the concentrations of dye at initial and equilibrium respectively, V (L): the volume of the solution and M (g): the mass of dry TiO_2_@HNTs photocatalyst used.

#### Photocatalytic PVC membrane activity test with MB dye

Photocatalytic PVC membrane TiO_2_@HNTs M2 and TiO_2_@HNTs M3 pieces (1 cm^2^ per piece) with control membrane were dipped into 20 ml aqueous dye solution of MB dye and kept in dark for1 h. Membrane pieces were then positioned in open petri dishes individually and kept in UV light (Philips 15 W UV light). Digital images of these membranes were captured after keeping them on petri plates in dark and after exposure to UV light.

## Supplementary information

Supply_TiO2@HNT/PVC Membrane
