# Supplementary material for: TiO2 decorated functionalized halloysite nanotubes (TiO2@HNTs) and photocatalytic PVC membranes synthesis, characterization and its application in water treatment
Source: Sci Rep. 2019 Mar 13;9:4345. doi: 10.1038/s41598-019-40775-4 (PMC6416328; doi:10.1038/s41598-019-40775-4)
Supplement: Supplementary file 1 — Supply_TiO2@HNT/PVC Membrane [file 41598_2019_40775_MOESM1_ESM.pdf]

Supporting Information for

TiO_2 decorated functionalized halloysite nanotubes (TiO_2@HNTs) and photocatalytic PVC membranes synthesis, characterization and its application in water treatment

Gourav Mishra and Mausumi Mukhopadhyay*

Department of Chemical Engineering, Sardar Vallabhbhai National Institute of Technology, Surat, Gujarat, India.

*Email: mausumi_mukhopadhyay@yahoo.com, mmu@ched.svnit.ac.in

Characterizations

Liquid chromatography–mass spectrometry (LC-MS)

**Agilent Technologies, USA, Column Agilent (G1316C)**			
**HiP Sampler (G4226A)**	**Binary Pump (G4220B):**	**Source parameter**	**TOF/Q-TOF Mass Spectrometer (G6550A)**
Draw speed: 100µL min^{-1}	Flow: 200µL min^{-1}	Nebulizer pressure (psig): 35	Ion source: Dual AJS ESI
Eject speed: 100µL min^{-1}	Solvent: Acetonitrile	Gas Temp. (◦C): 250	
Injection volume: 8µL		Gas flow rate (L min^{-1}): 13	
		Sheath Gas Temp. (◦C): 300	
		Sheath Gas flow (L min^{-1}): 11	

Instrument Details: The chromatographic experiments with LCMS system were carried out on an Agilent 1290 Infinity UHPLC System, 1260 infinity Nano HPLC with Chipcube, 6550 iFunnel Q-TOFs (Agilent Technologies, USA) with a Column, binary pump and an autosampler. Acetonitrile was used as mobile phase solvent. The mass spectrometer was equipped with an electrospray ionization (ESI) source. The mass range was from 50 to 1000 *m/z*. Degradation products were monitored by LC-MS.

- Column Details:- Zorbax eclipse c18, 2.1 x 150mm 5-micron

Acquisition Method Report

Acquisition Method Info

**Method Name**	30mins_+ESI_10032014_MSMS.m
**Method Path**	D:\MassHunter\methods\30mins_+ESI_10032014_MSMS.m
**Method Description**	Default Method

Device List

- HiP Sampler
- Binary Pump
- Column Comp.
- Q-TOF

TOF/Q-TOF Mass Spectrometer

**Component Name**	MS Q-TOF	**Component Model**	G6550A
**Ion Source**	Dual AJS ESI	**Stop Time (min)**	No Limit/As Pump
**Can wait for temp.**	Enable	**Fast Polarity**	N/A
**MS Abs. threshold**	200	**MS Rel. threshold(%)**	0.010
**MS/MS Abs. threshold**	5	**MS/MS Rel. threshold(%)**	0.010
**Tune File**	Autotune.tun		

Time Segments

Time Segment #	Start Time (min)	Diverter Valve State	Storage Mode	Ion Mode
1	0	MS	Both	Dual AJS ESI

Time Segment 1

Acquisition Mode AutoMS2

MS Min Range (m/z)	50
MS Max Range (m/z)	1000
MS Scan Rate (spectra/sec)	1.00
MS/MS Scan Rate (spectra/sec)	1.00
Isolation Width MS/MS	Medium (~4 amu)

Ramped Collision Energy

Charge	Slope	Offset
1	3.6	-2.6
2	3.6	-2.6

Precursor Selection

Max Precursors Per Cycle	8
Threshold (Abs)	5000
Threshold (Rel)(%)	0.010
Precursor abundance based scan speed	Yes
Target (counts/spectrum)	25000.000
Use MS/MS accumulation time limit	Yes
Use dynamic precursor rejection	No
Purity Stringency (%)	100.000
Purity Cutoff (%)	30.000
Isotope Model	Common
Active exclusion enabled	Yes
Active exclusion excluded after (spectra)	1
Active exclusion released after (min)	0.20
Sort precursors	By abundance only

Charge State Preference

Selected Charges
1
2
Unk

Source Parameters

Parameter	Value
Gas Temp (°C)	250
Gas Flow (l/min)	13
Nebulizer (psig)	35
SheathGasTemp	300
SheathGasFlow	11

Scan Segments

Scan Seg #	Ion Polarity
1	Positive

Scan Segment 1

Scan Source Parameters

Parameter	Value
VCap	3500
Nozzle Voltage (V)	1000
Fragmentor	175
Skimmer1	65
OctopoleRFPeak	750

ReferenceMasses

Ref Mass Enabled	Disabled
Ref Nebulizer (psig)	

Chromatograms

Chrom Type	Label	Offset	Y-Range
TIC	TIC	15	10000000

Name: HiP Sampler — Model: G4226A

**Auxiliary**	
Draw Speed	100.0 µL/min
Eject Speed	100.0 µL/min
Draw Position Offset	0.0 mm
Wait Time After Drawing	2.0 s
Sample Flush Out Factor	5.0
Vial/Well bottom sensing	Yes
**Injection**	
Injection Mode	Injection with needle wash
Injection Volume	8.00 µL
**Needle Wash**	
Needle Wash Location	Flush Port
Wash Time	3.0 s
**High throughput**	
Automatic Delay Volume Reduction	No
**Overlapped Injection**	
Enable Overlapped Injection	No
**Valve Switching**	
Valve Movements	0
**Valve Switch Time 1**	
Switch Time 1 Enabled	Yes
Switch Time 1	0.01 min
**Valve Switch Time 2**	
Switch Time 2 Enabled	No
**Valve Switch Time 3**	
Switch Time 3 Enabled	No
**Valve Switch Time 4**	
Switch Time 4 Enabled	No
**Stop Time**	
Stoptime Mode	As pump/No limit
**Post Time**	
Posttime Mode	Off

Name: Binary Pump — Model: G4220B

Flow	0.200 mL/min
Use Solvent Types	Yes
Stroke Mode	Synchronized
Low Pressure Limit	0.00 bar
High Pressure Limit	1200.00 bar
Max. Flow Ramp Up	100.000 mL/min²
Max. Flow Ramp Down	100.000 mL/min²
Expected Mixer	No check
**Stroke A**	
Automatic Stroke Calculation A	Yes
**Compress A**	
Compressibility Mode A	Compressibility Value Set
Compressibility A	45 10e-6/bar
**Compress B**	
Compressibility Mode B	Compressibility Value Set
Compressibility B	75 10e-6/bar
**Stop Time**	
Stoptime Mode	Time set
Stoptime	30.00 min
**Post Time**	
Posttime Mode	Time set
Posttime	2.00 min

Timetable

Timetable

	Time	Function	Parameter
1	2.00 min	Change Solvent Composition	Solvent composition A: 95.00 % B:5.00 %
2	2.00 min	Change Flow	Flow: 0.2 mL/min
3	2.00 min	Change Max. Pressure Limit	Max. Pressure Limit: 1200.00 bar
4	20.00 min	Change Solvent Composition	Solvent composition A: 5.00 % B:95.00 %
5	20.00 min	Change Flow	Flow: 0.2 mL/min
6	20.00 min	Change Max. Pressure Limit	Max. Pressure Limit: 1200.00 bar
7	25.00 min	Change Solvent Composition	Solvent composition A: 5.00 % B:95.00 %
8	25.00 min	Change Flow	Flow: 0.2 mL/min
9	25.00 min	Change Max. Pressure Limit	Max. Pressure Limit: 1200.00 bar
10	26.00 min	Change Solvent Composition	Solvent composition A: 95.00 % B:5.00 %
11	26.00 min	Change Flow	Flow: 0.2 mL/min
12	26.00 min	Change Max. Pressure Limit	Max. Pressure Limit: 1200.00 bar
13	30.00 min	Change Solvent Composition	Solvent composition A: 95.00 % B:5.00 %
14	30.00 min	Change Flow	Flow: 0.2 mL/min
15	30.00 min	Change Max. Pressure Limit	Max. Pressure Limit: 1200.00 bar

Solvent Composition

	Channel	Ch. 1 Solv.	Name 1	Ch2 Solv.	Name 2	Selected	Used	Percent
1	A	100.0 % Water V.02	0.1% FA in water	100.0 % Water V.02		Ch. 1	Yes	95.00 %
2	B	100.0 % Acetonitrile V.02	90% ACN +10% H2O+ 0.1% FA	100.0 % Acetonitrile V.02		Ch. 1	Yes	5.00 %

Name: Column Comp. Model: G1316C

Ready when front door open	Yes
**Left Temperature Control**	
Temperature Control Mode	Not Controlled
**Enable Analysis Left Temperature**	
Enable Analysis Left Temperature On	Yes
Enable Analysis Left Temperature Value	0.80 °C
**Right Temperature Control**	
Right temperature Control Mode	Temperature Set
Right temperature	25.00 °C
**Enable Analysis Right Temperature**	
Enable Analysis Right Temperature On	Yes
Enable Analysis Right Temperature Value	0.80 °C
**Stop Time**	
Stoptime Mode	As pump/injector
**Post Time**	
Posttime Mode	Off

Result and discussion

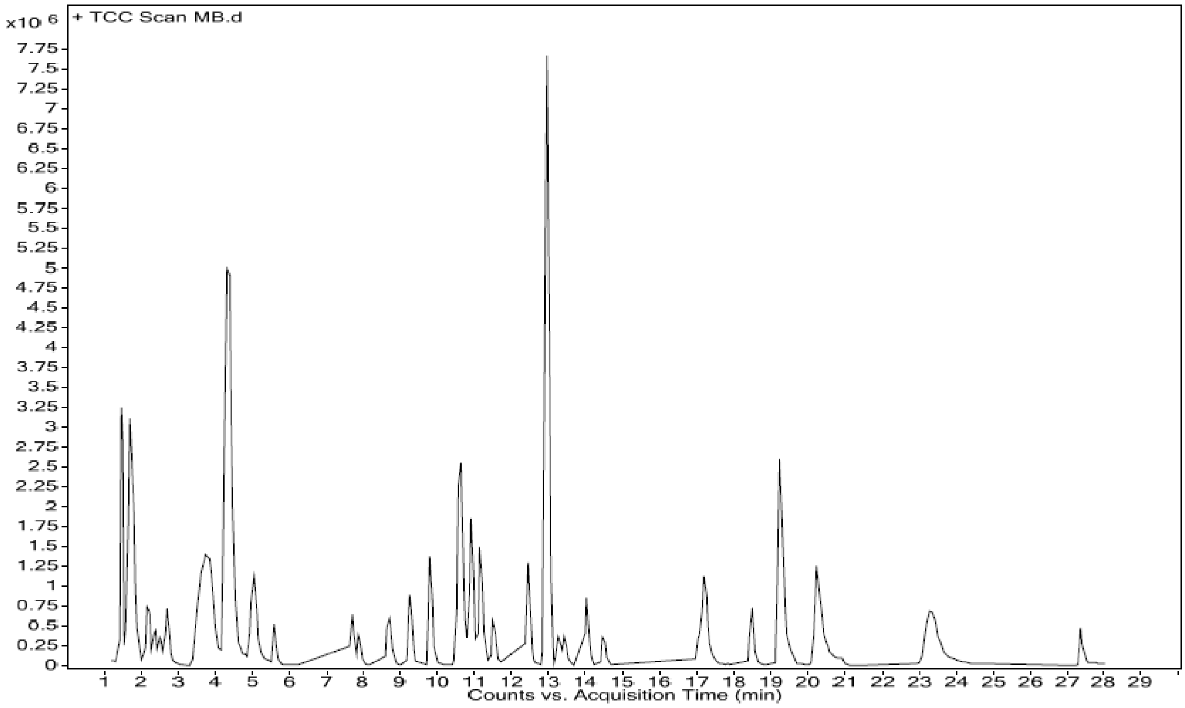

Figure S1. Chromatogram of the MB dye degradation solution.

Table S2 Mass spectra and the possible structure of the MB dye degradation products.

S.No. | **Spectra** | **Structure**

1. **$C_2H_7NO_3S$**

m/z	RT	Mass	DB	DB diff
129.99	1.48	125.01	1	7.27

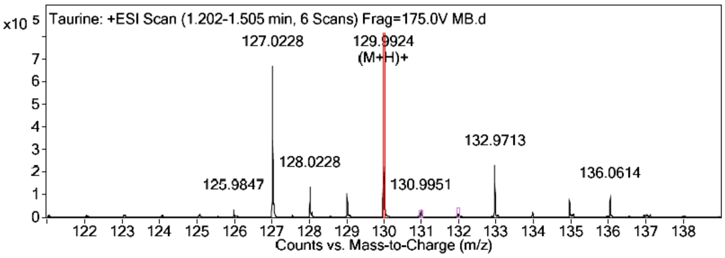

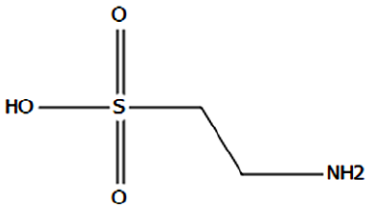

2. **$C_{12}H_{10}O_3$**

m/z	RT	Mass	DB	DB diff
185.06	1.48	202.06	1	-22.17

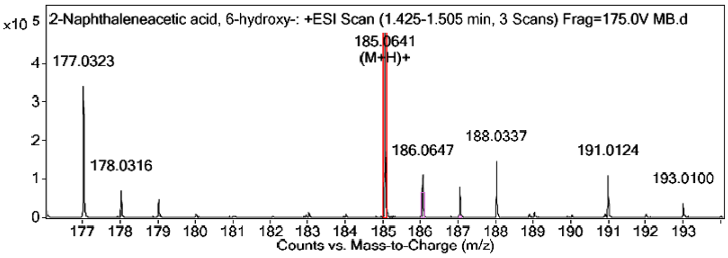

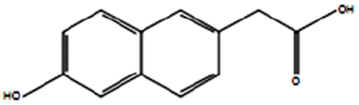

3. $C_5H_{14}NO$

m/z	RT	Mass	DB	DB diff
104.10	1.66	104.10	2	-23.07

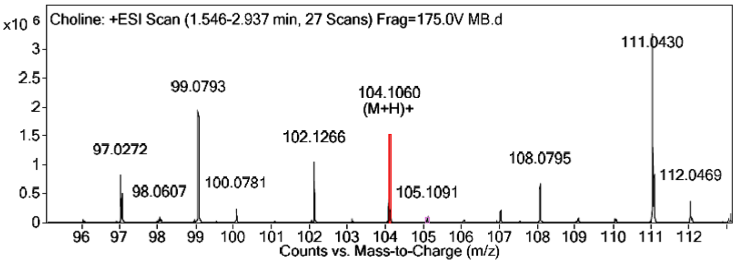

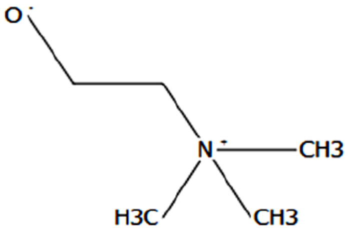

4. **$C_7H_{16}NO_3$**

m/z	RT	Mass	DB	DB diff
162.11	1.71	162.11	2	9.34

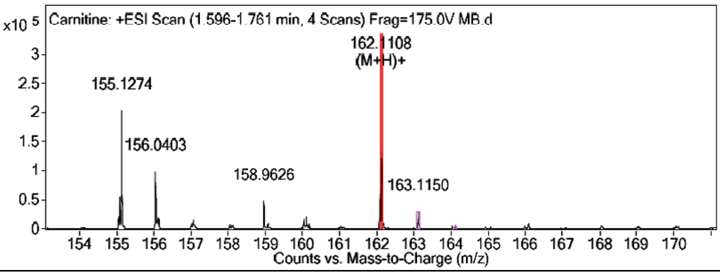

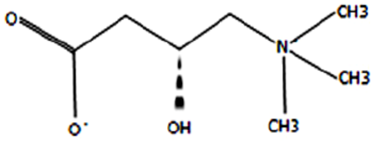

5. **$C_6H_{12}O_6$**

m/z	RT	Mass	DB	DB diff
203.05	1.71	180.06	15	10.39

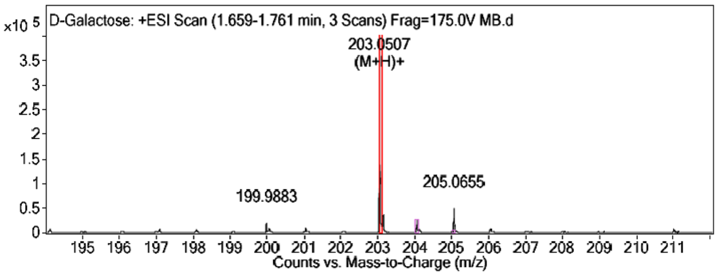

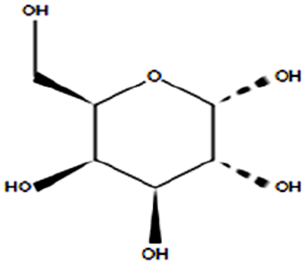

6. **$C_5H_{12}NO_2$**

m/z	RT	Mass	DB	DB diff
118.08	1.72	118.08	9	9.98

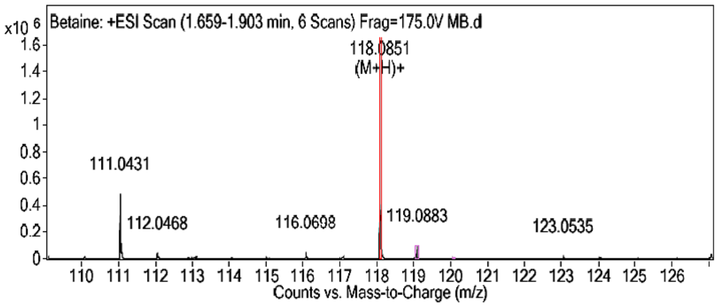

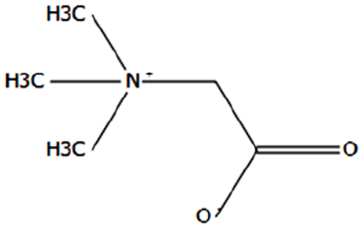

7. **$C_6H_6N_2O_2$**

m/z	RT	Mass	DB	DB diff
121.03	2.37	138.04	5	8.24

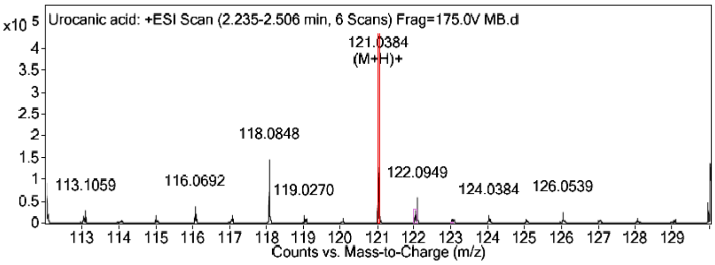

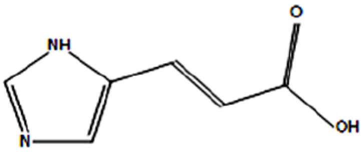

8. **$C_4H_8N_2O_2$**

m/z	RT	Mass	DB	DB diff
139.04	2.18	116.05	7	-8.24

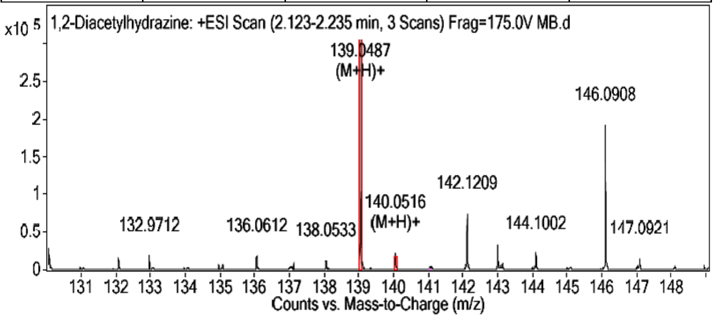

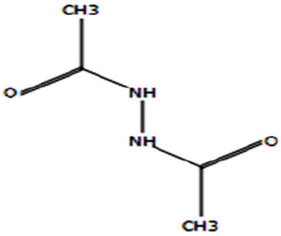

9. **$C_5H_9NO_4$**

m/z	RT	Mass	DB	DB diff
130.04	2.52	14.05	10	9.01

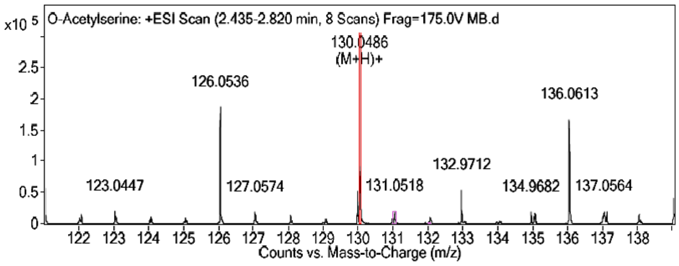

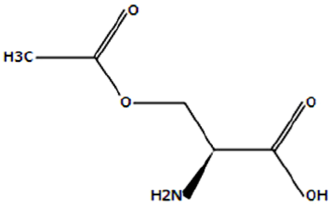

10. **$C_9H_{19}NO_2$**

m/z	RT	Mass	DB	DB diff
156.13	2.69	173.14	2	9.13

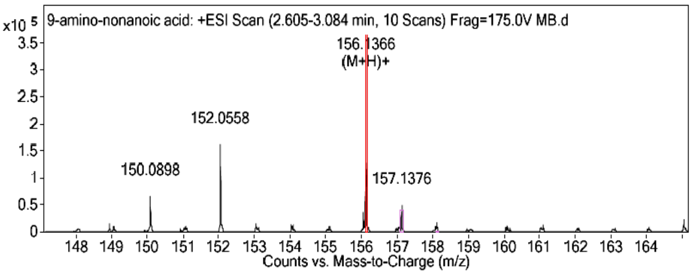

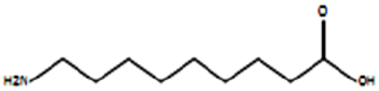

11. **$C_6H_{14}N_2O_3$**

m/z	RT	Mass	DB	DB diff
167.07	2.72	162.10	5	-3.75

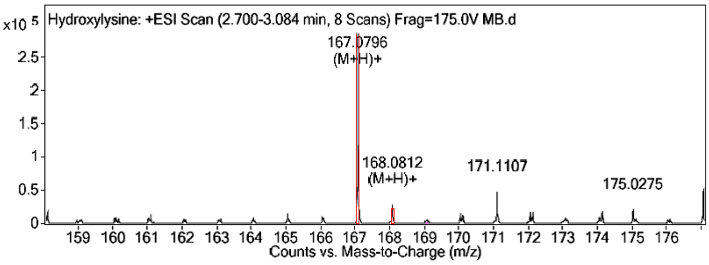

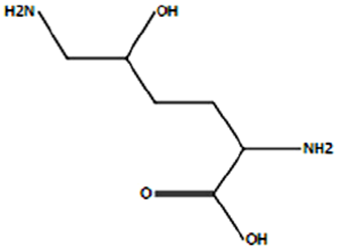

12. **$C_7H_{15}NO_2$**

m/z	RT	Mass	DB	DB diff
168.10	4.35	145.11	14	-3.29

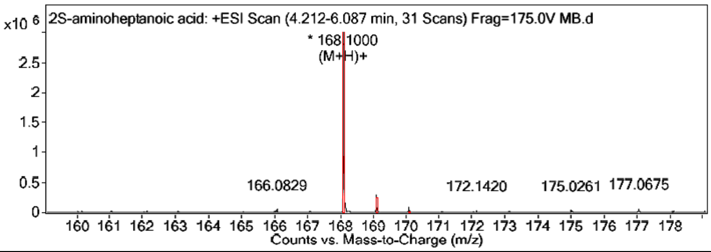

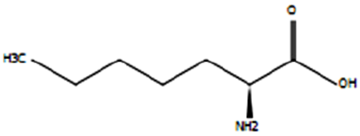

13. **$C_{11}H_{14}ClNO$**

m/z	RT	Mass	DB	DB diff
234.06	7.70	211.07	3	-7.87

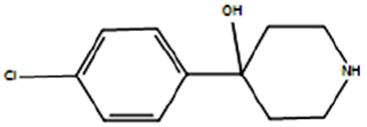

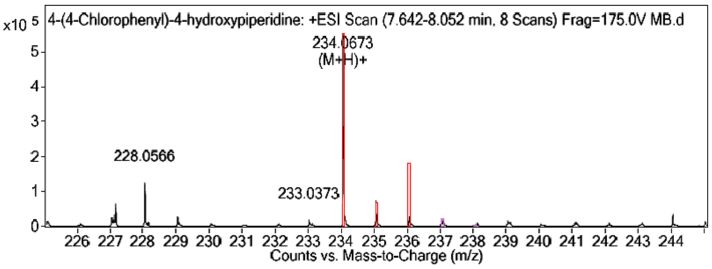

14. $C_6H_8N_2O_2S$

m/z	RT	Mass	DB	DB diff
195.02	7.88	172.03	3	-1.69

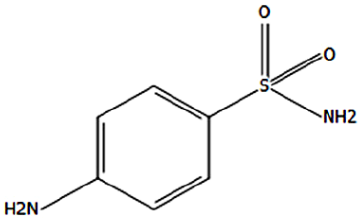

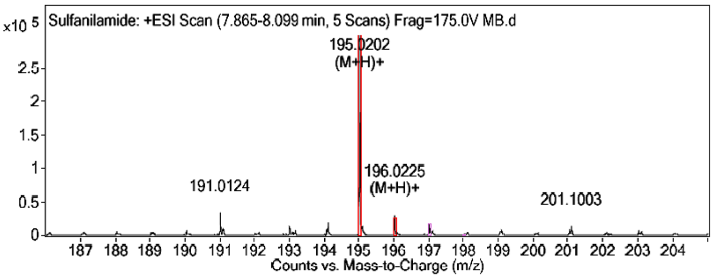

15. **$C_{10}H_{17}N_3O_2S$**

m/z	RT	Mass	DB	DB diff
248.08	8.65	243.10	8	-0.48

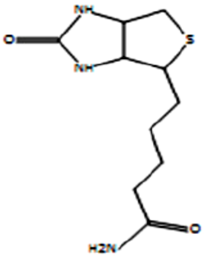

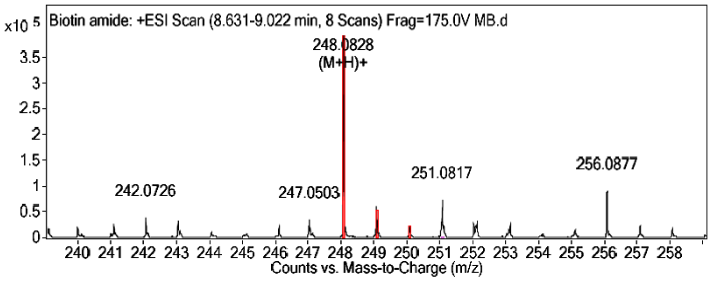

16. **$C_{12}H_{13}N_3O_4S$**

m/z	RT	Mass	DB	DB diff
278.08	8.74	295.06	8	8.6

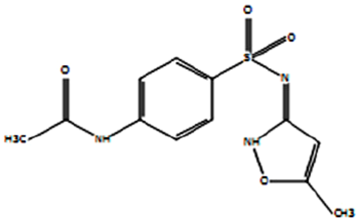

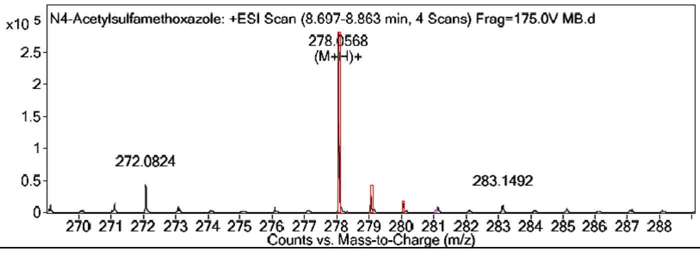

17. **$C_{13}H_{15}N_3O_4S$**

m/z	RT	Mass	DB	DB diff
292.07	9.27	309.07	11	8.23

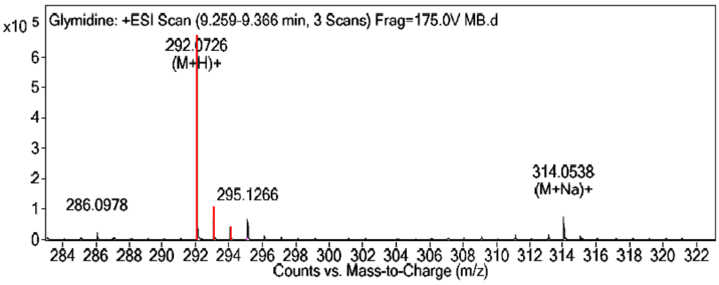

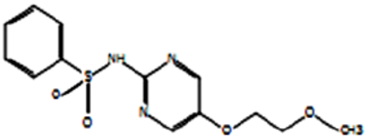

18. **$C_6H_8N_2O_4$**

m/z	RT	Mass	DB	DB diff
155.04	10.60	172.04	15	-0.18

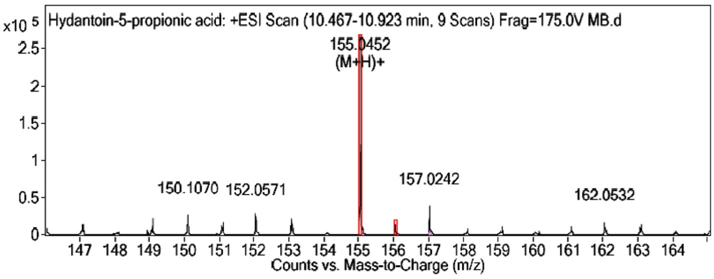

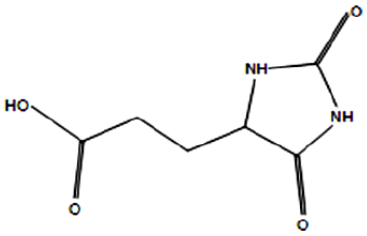

19. **$C_{10}H_{13}N_3OS$**

m/z	RT	Mass	DB	DB diff
246.06	13.27	223.07	4	-0.35

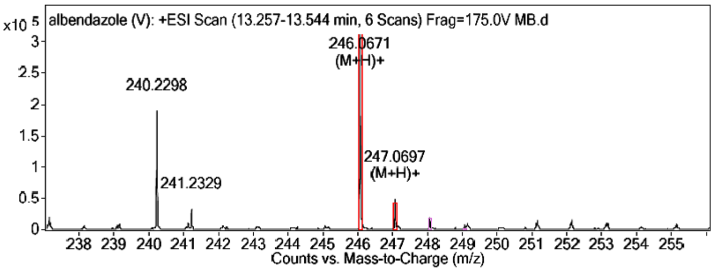

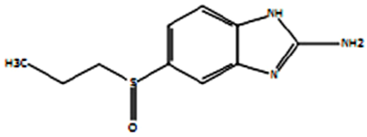

20. **$C_{18}H_{36}O_5$**

m/z	RT	Mass	DB	DB diff
355.24	17.22	332.25	13	8.97

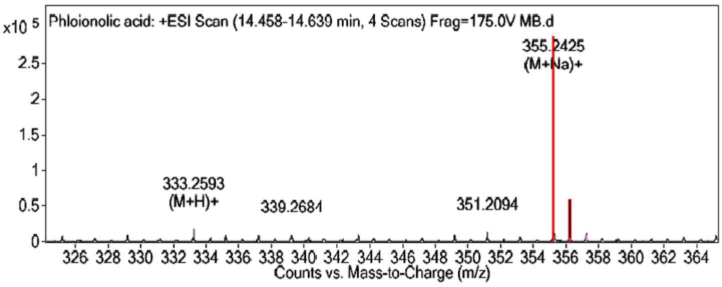

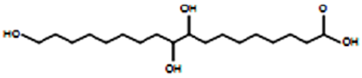

21. **$C_{16}H_{32}O_2$**

m/z	RT	Mass	DB	DB diff
279.22	17.22	256.24	15	0.22

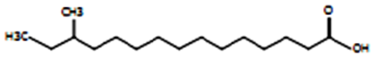

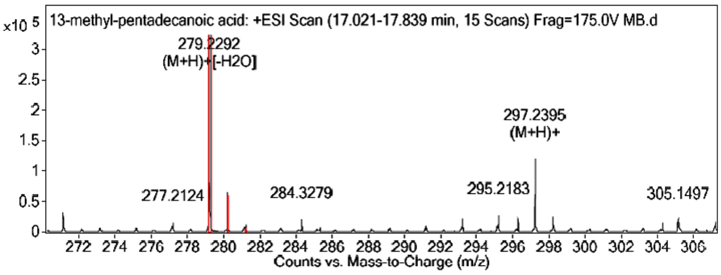

22. **$C_6H_{14}N_2O$**

m/z	RT	Mass	DB	DB diff
113.10	27.36	130.11	1	-1.31

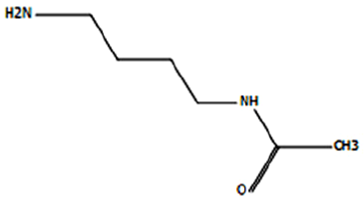

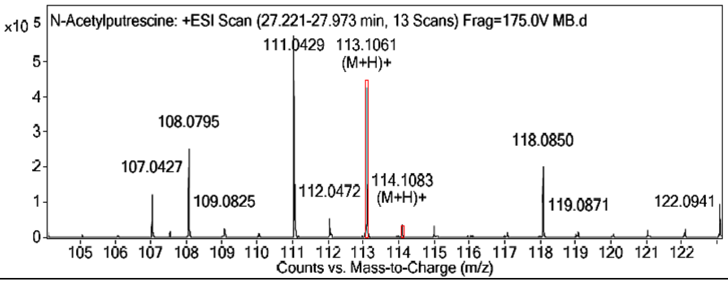

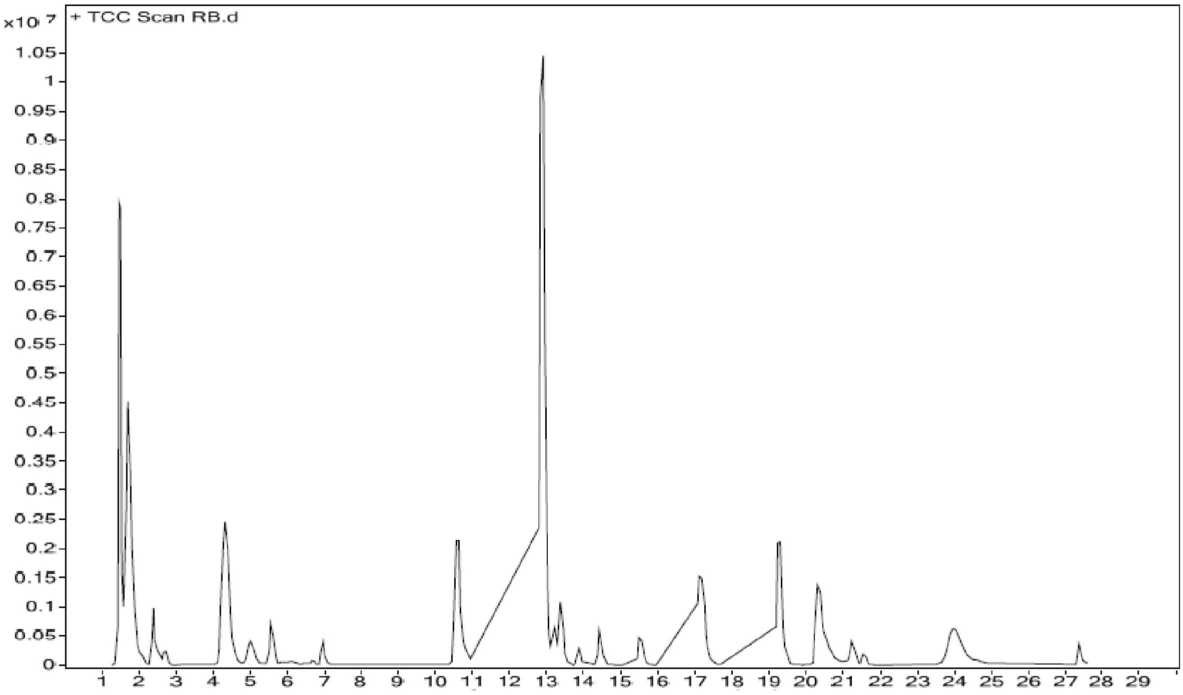

Figure S2. Chromatogram of the RB dye degradation solution.

Table S3 Mass spectra and the possible structure of the RB dye degradation products.

S.No.	Spectra	Structure
1.	**$C_{12}H_{10}O_3$**	

m/z	RT	Mass	DB	DB diff
185.06	1.48	202.06	1	-15.09

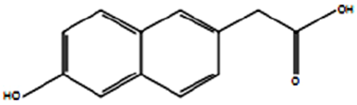

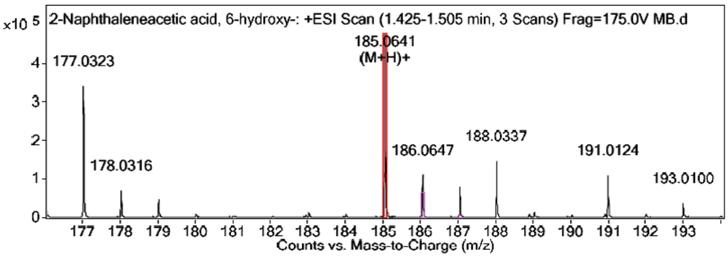

S.No.	Spectra	Structure
2.	**$C_5H_{14}NO$**	

m/z	RT	Mass	DB	DB diff
104.10	1.64	104.10	2	-12.13

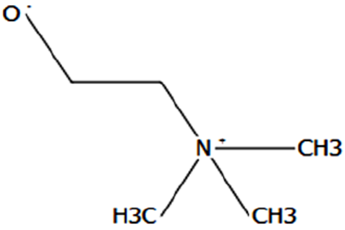

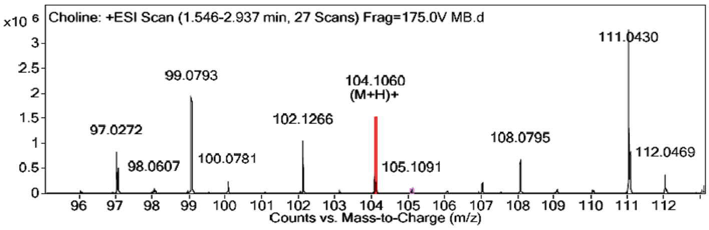

3. **$C_7H_{16}NO_3$**

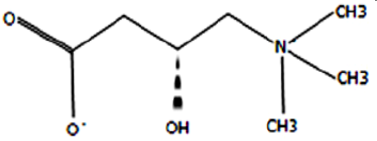

m/z	RT	Mass	DB	DB diff
144.09	1.81	162.11	4	18.21

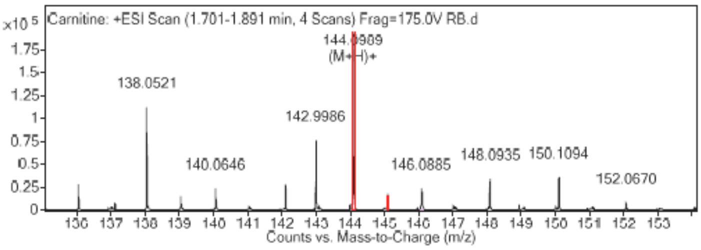

4. **C_3H_7NO**

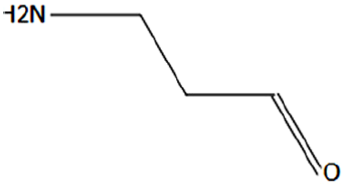

m/z	RT	Mass	DB	DB diff
78.03	1.47	73.05	3	13.09

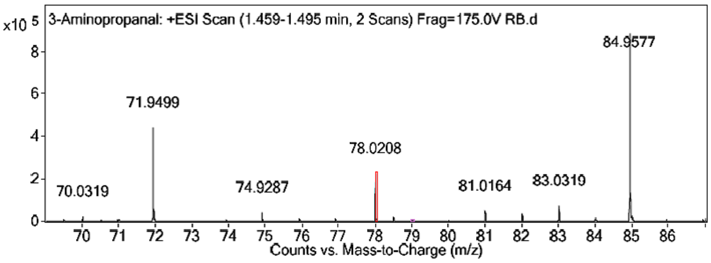

5. **$C_5H_{12}NO_2$**

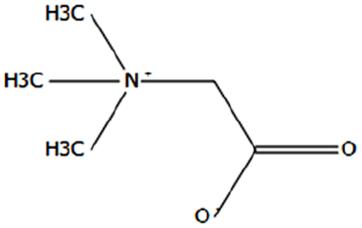

m/z	RT	Mass	DB	DB diff
118.08	1.72	118.08	9	19.93

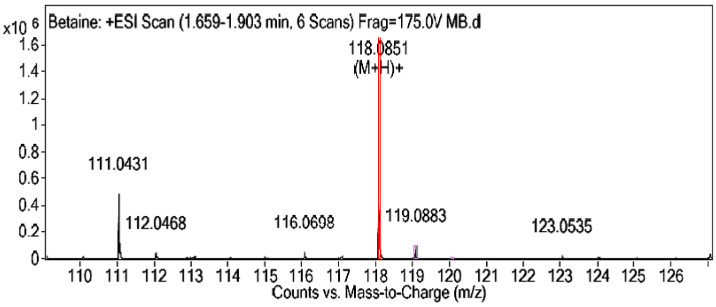

6. **$C_5H_{11}NO_4$**

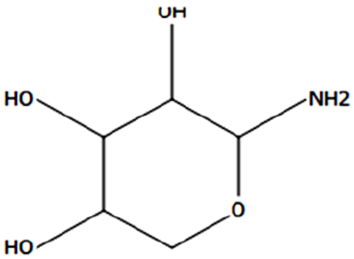

m/z	RT	Mass	DB	DB diff
172.05	1.47	149.06	1	24.71

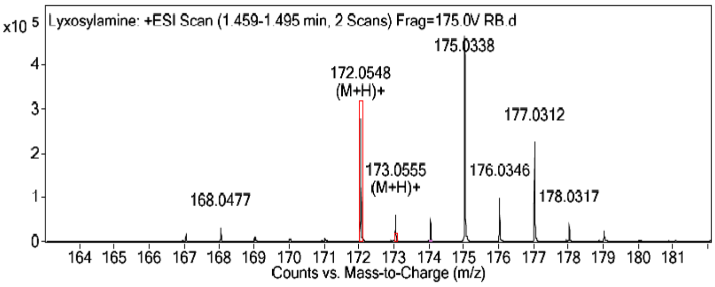

7. **$C_4H_8N_2O_2$**

m/z	RT	Mass	DB	DB diff
99.05	2.38	116.06	5	-19.93

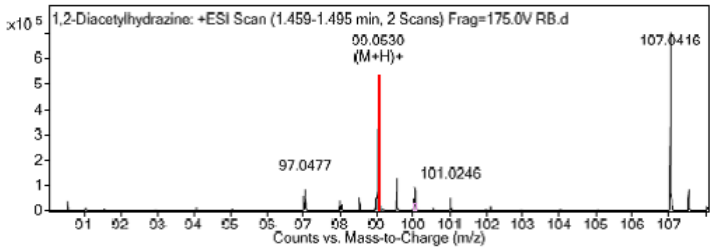

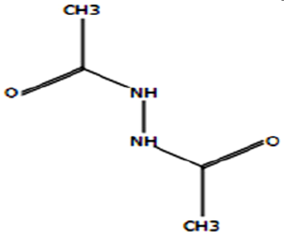

8. **$C_5H_9NO_4$**

m/z	RT	Mass	DB	DB diff
130.04	2.54	147.05	10	17.36

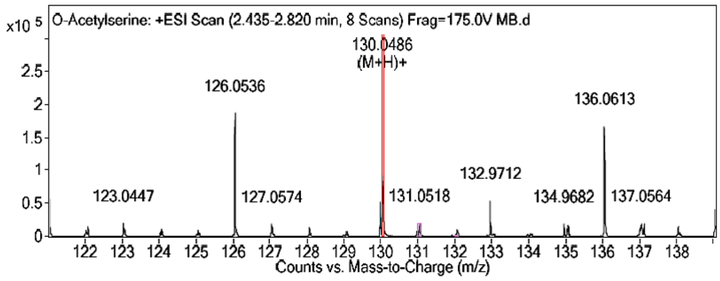

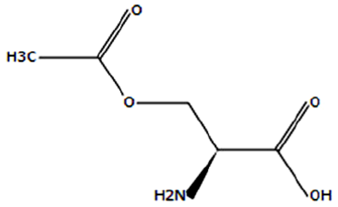

9. **$C_5H_{10}O_2$**

m/z	RT	Mass	DB	DB diff
107.04	1.74	102.06	4	23.82

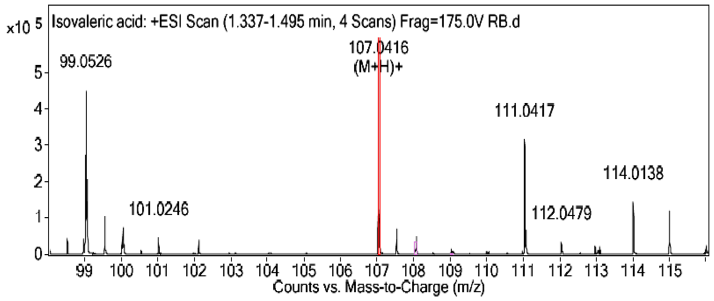

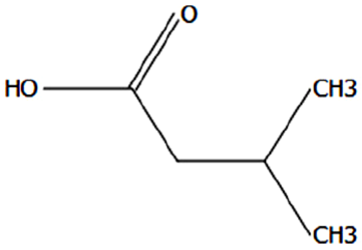

10. **$C_7H_{15}NO_2$**

m/z	RT	Mass	DB	DB diff
168.09	4.33	145.10	14	5.26

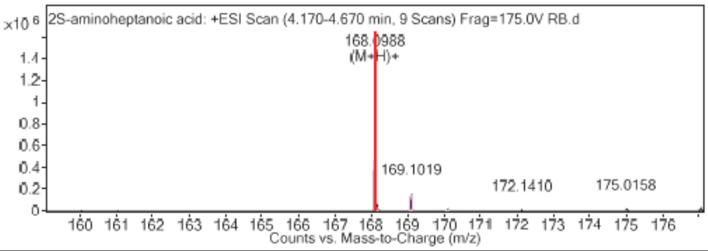

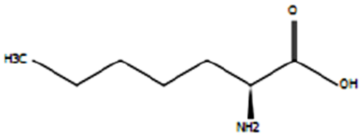

11. $C_6H_{14}N_2O_3$

m/z	RT	Mass	DB	DB diff
167.07	2.70	162.09	4	5.22

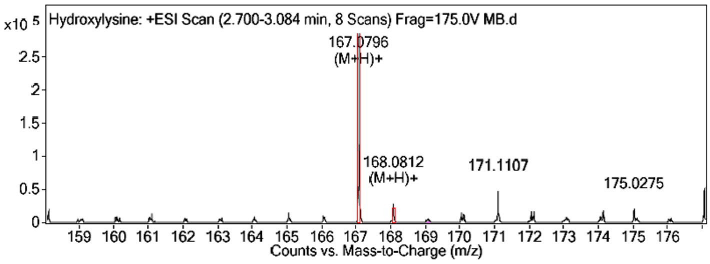

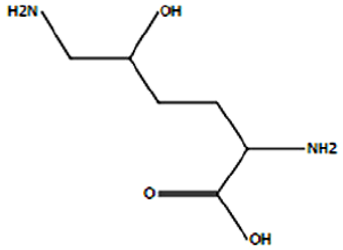

12. $C_{18}H_{20}O_3$

m/z	RT	Mass	DB	DB diff
289.12	6.975	284.14	5	-3.85

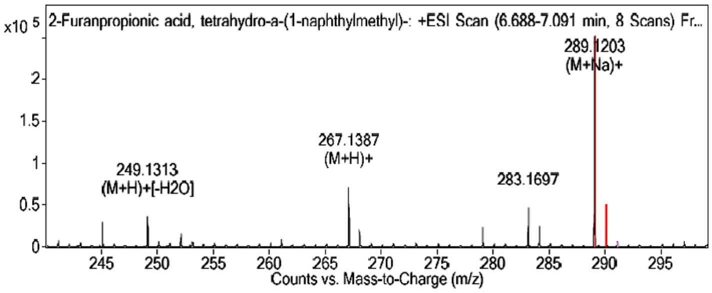

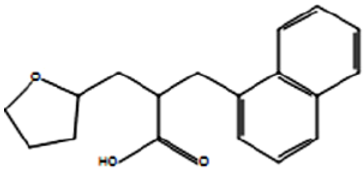

13. $C_4H_8N_2O_3$

m/z	RT	Mass	DB	DB diff
155.04	10.58	132.05	15	-6.94

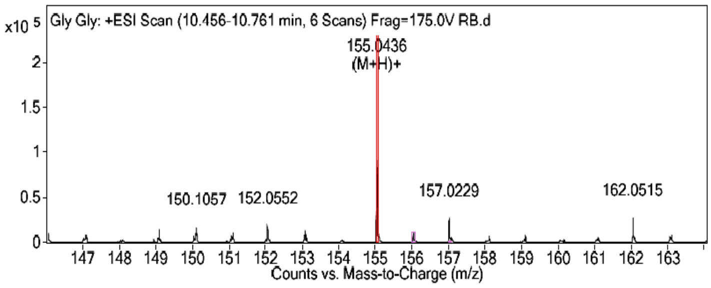

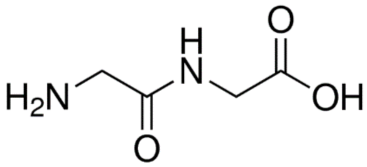

14. $C_6H_8O_4$

m/z	RT	Mass	DB	DB diff
149.02	20.33	144.04	8	4.67

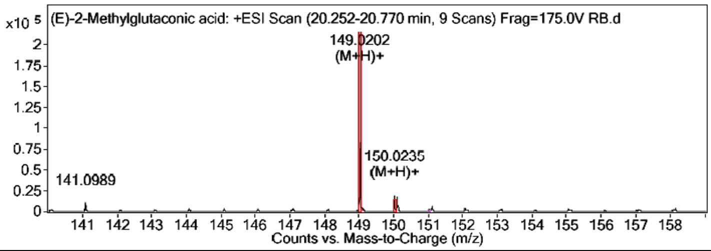

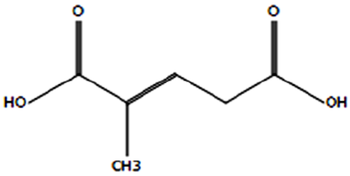

15. **$C_{18}H_{36}O_5$**

m/z	RT	Mass	DB	DB diff
337.23	17.25	332.25	13	15.11

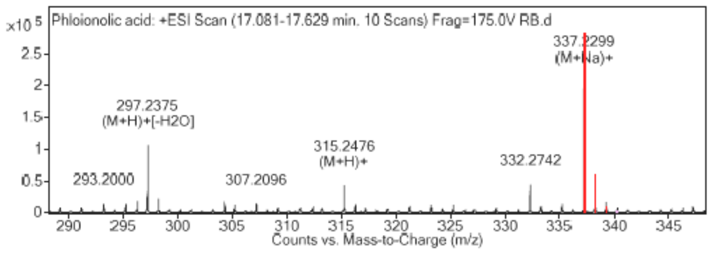

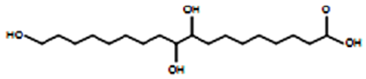

16. **$C_{16}H_{32}O_2$**

m/z	RT	Mass	DB	DB diff
279.22	17.25	256.23	15	8.9

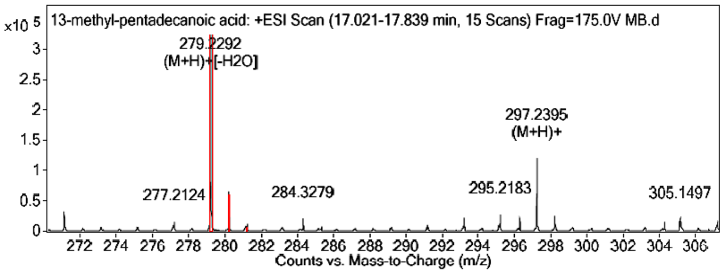

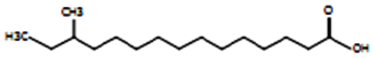

17. **$C_6H_{14}N_2O$**

m/z	RT	Mass	DB	DB diff
113.10	27.36	130.10	1	8.64

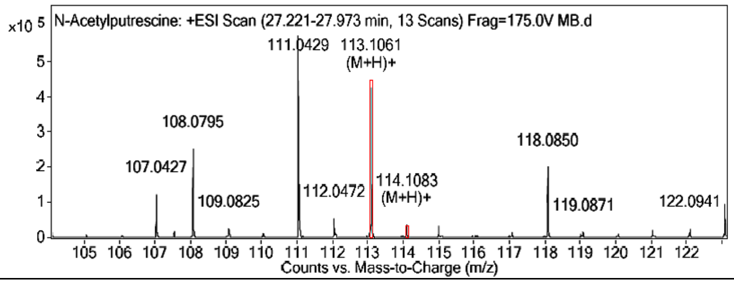

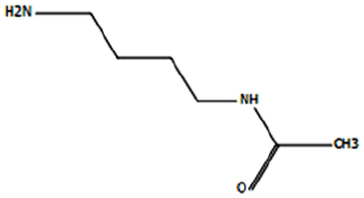

Membrane stability Test

The stability of TiO_2 NPs attached to HNTs in the membranes was also investigated for any loss in TiO_2 NPs from the membranes. The membrane sample (1.0 g) was cut into pieces and immersed in 500 mL of deionized water with continuous stirring at room temperature. Three hours later, the water sample was collected and additional 500 mL of deionized water was added, and the steps were repeated. The concentrations of titanium ions were analyzed with an Inductively coupled plasma atomic emission spectroscopy (ICP AES) (SPECTRO Analytical Instruments GmbH, Germany) after regular interval.

The immobilized TiO_2@HNTs NPs on the membranes were not leached out after 48 hr experiment (less than 0.01 ppm concentration detected). The results indicate that the leaching of TiO_2 NPs was not found and hence TiO_2 is not a threat for secondary pollution (Ray et al.2009).

Reference

Ray, P. C., Yu, H. & Fu, P. P. Toxicity and environmental risks of nanomaterials: challenges and future needs. *Journal of Environmental Science and Health Part C* **27**, 1-35 (2009).

Table S4 Permeation properties of the photocatalytic PVC membranes[44]

Membrane	Porosity Ɛ (%)	Pore size (nm)	Water contact angle (^{0})	Pure water flux ($Lm^{-2}h^{-1}$)
TiO_2@HNTs 0	42.80	25.04 ±1.4	82.44±7.3	109.1±9.6
TiO_2@HNTs 2	52.39	30.00 ± 0.5	69.63±4.3	197.5±3.6
TiO_2@HNTs 3	44.49	28.77 ± 0.2	68.87±5.6	179.2±3.3

Reference

[44] Mishra, G. & Mukhopadhyay, M. Flux improvement, rejection, surface energy and antibacterial properties of synthesized TiO_2-Mo.HNTs/PVC nanocomposite ultrafiltration membranes. *New Journal of Chemistry* **41**, 15049-15057
